# Increased Apoptosis of Myoblasts in *Drosophila* Model for the Walker-Warburg Syndrome

**DOI:** 10.1371/journal.pone.0011557

**Published:** 2010-07-13

**Authors:** Morio Ueyama, Yoshihiro Akimoto, Tomomi Ichimiya, Ryu Ueda, Hayato Kawakami, Toshiro Aigaki, Shoko Nishihara

**Affiliations:** 1 Department of Bioinformatics, Soka University, Hachioji, Tokyo, Japan; 2 Department of Anatomy, Kyorin University School of Medicine, Mitaka, Tokyo, Japan; 3 Invertebrate Genetics Laboratory, National Institute of Genetics, Mishima, Shizuoka, Japan; 4 Department of Biological Science, Tokyo Metropolitan University, Hachioji, Tokyo, Japan; Brigham and Women's Hospital, Harvard Medical School, United States of America

## Abstract

Walker-Warburg syndrome, a progressive muscular dystrophy, is a severe disease with various kinds of symptoms such as muscle weakness and occasional seizures. The genes of protein *O*-mannosyltransferases 1 and 2 (*POMT1* and *POMT2*), fukutin, and fukutin-related protein are responsible for this syndrome. In our previous study, we cloned *Drosophila* orthologs of human *POMT1* and *POMT2* and identified their activity. However, the mechanism of onset of this syndrome is not well understood. Furthermore, little is known about the behavioral properties of the *Drosophila POMT1* and *POMT2* mutants, which are called *rotated abdomen* (*rt*) and *twisted* (*tw*), respectively. First, we performed various kinds of behavioral tests and described in detail the muscle structures by using these mutants. The mutant flies exhibited abnormalities in heavy exercises such as climbing or flight but not in light movements such as locomotion. Defective motor function in mutants appeared immediately after eclosion and was exaggerated with aging. Along with motor function, muscle ultrastructure in the *tw* mutant was altered, as seen in human patients. We demonstrated that expression of RNA interference (RNAi) for the *rt* gene and the *tw* mutant was almost completely lethal and semi-lethal, respectively. Flies expressing RNAi had reduced lifespans. These findings clearly demonstrate that *Drosophila POMT* mutants are models for human muscular dystrophy. We then observed a high density of myoblasts with an enhanced degree of apoptosis in the *tw* mutant, which completely lost enzymatic activity. In this paper, we propose a novel mechanism for the development of muscular dystrophy: *POMT* mutation causes high myoblast density and position derangement, which result in apoptosis, muscle disorganization, and muscle cell defects.

## Introduction

Congenital muscular dystrophies (CMDs) are genetic diseases that cause progressive muscle weakness and wasting [1], [2]. CMDs result from dystrophin glycoprotein complex (DGC) dysfunction [3]. DGC, which connects the extracellular matrix to the intracellular cytoskeleton, comprises several kinds of proteins such as laminin 2, dystrophin, sarcoglycan, and dystroglycan [4].

Walker-Warburg Syndrome (WWS), the most severe CMD, is a rare recessive inherited disorder characterized by muscular dystrophy, severe brain malformations, and eye abnormalities [5]–[9]. Patients with WWS rarely survive to birth, and even if they do, the chances that they will survive to adulthood are low [10].

The genes of protein *O*-mannosyltransferase 1 and 2 (*POMT1* and *POMT2*), fukutin (*FCMD*), and fukutin-related protein (*FKRP*) are responsible for WWS [11]–[17]. The POMT1/2 complex transfers mannose to the Ser/Thr residues of α-dystroglycan [18], one of the components of the DGC, and plays an important role in the first step of *O*-mannosylation. *O*-Mannosylation contributes to the stabilization of sarcolemma by binding to laminin, which attaches to the basal membrane [3], [19]–[21].

Recently, several mutations were found in the *POMT1* and *POMT2* genes of WWS patients [11]–[15]. These mutations cause a reduction in *O*-mannosylation of α-dystroglycan, which results in WWS. In fact, recombinant mutant forms of POMT1 co-expressed with wild-type POMT2 did not show any *O*-mannosyltransferase activity [22].

Although a mouse model for WWS has been generated by targeted disruption of the *Pomt1* gene, the mouse ortholog of *POMT1*, the adult phenotype is unknown because *Pomt1* knockout mice are embryonic lethal [23].

In *Drosophila*, few studies on muscular dystrophy have been reported [24]–[26]. The *Drosophila* genome also has the components of DGC [27], [28]. The *Drosophila* orthologs of human *POMT1* and *POMT2* are called *rotated abdomen* (*rt*) and *twisted* (*tw*), respectively [29], [30]. In our previous study, we cloned genes of these orthologs and identified their activity. Their enzymatic activities are similar to those of the human enzymes; when both RT and TW were co-expressed in cultured insect cells, *O*-mannosyltransferase activity was observed [29]. Moreover, defective muscles and/or thin muscles with large sarcomeres were observed in larvae of *rt* or *tw* mutants [25], [31], and the synaptic transmission in larvae was also abnormal with changes in the subunit composition of the postsynaptic glutamate receptors at the neuromuscular junction [26].

In the current paper, we first analyze the behavioral properties and ultrastructure of adult muscles in *rt* and/or *tw* mutants and then provide evidence that these mutants are highly useful for elucidating the mechanism of muscular dystrophy. Finally, we report that the number of apoptotic myoblasts increases in *tw* mutants and propose a new mechanism for the development of muscular dystrophy, which involves an increase in the number of apoptotic myoblasts, thereby causing muscle disorganization.

## Results

### Behavioral defects in *rt* and *tw* mutants

**Figure 1:**
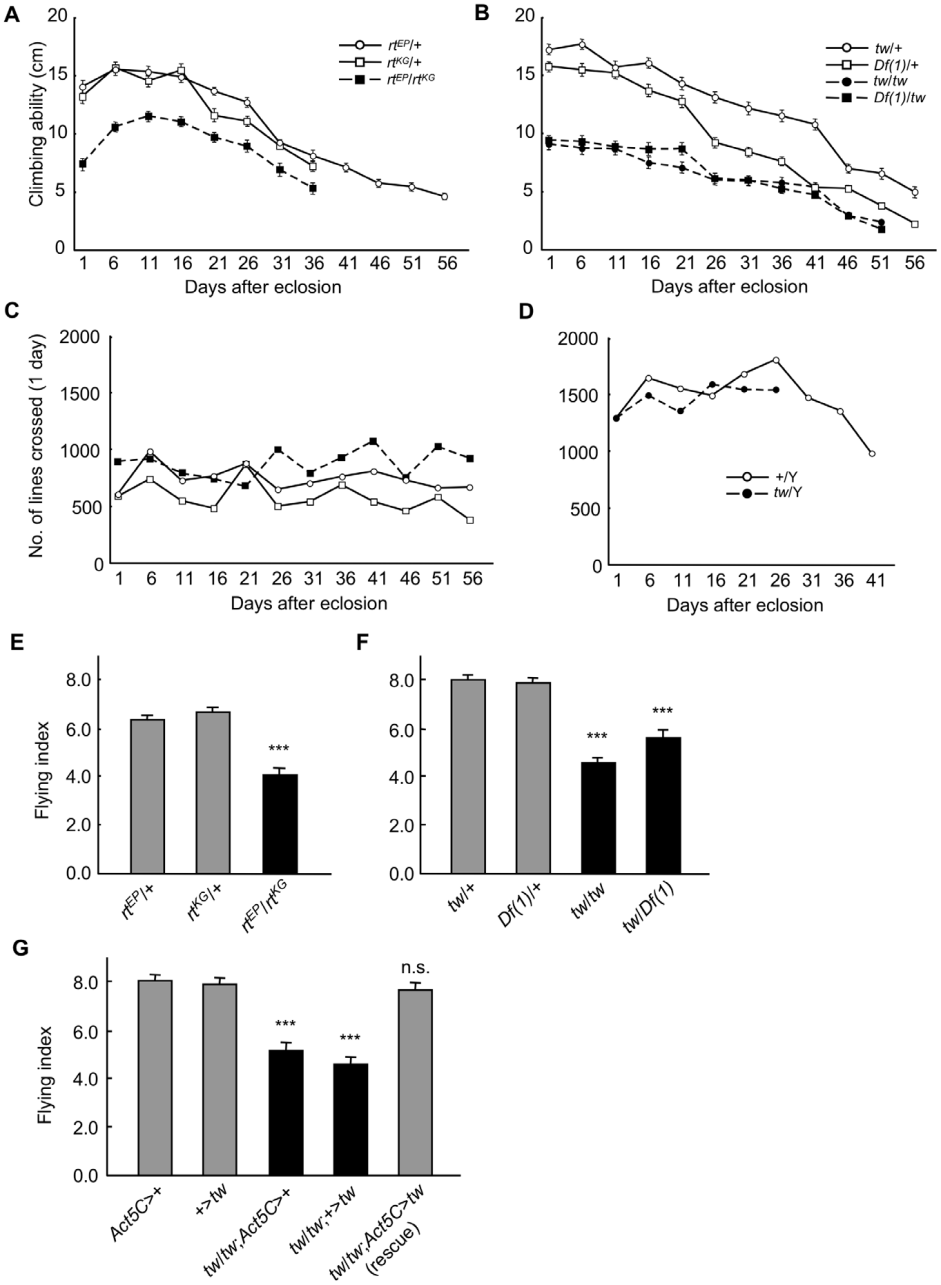
Behavioral defects in *rt* and *tw* mutant flies. (A) Age-related change in climbing ability in *rt* mutants (A) and *tw* mutants (B). Age-related change in locomotive activity in *rt* mutants (C) and *tw* mutants (D). Flying ability in *rt* mutants (E) and *tw* mutants (F) at 30–35 days after eclosion. Rescue of flying ability in *tw* mutants (G). Error bars in all figures indicate standard error. Results of statistical analyses in (A) and (B) are shown in Tables 1 and 2, respectively. ***p<0.001 by Tukey test. n.s., not significant.

Patients with progressive muscular dystrophy show muscle weakness and motor dysfunction with age. Therefore, we evaluated the motor function in *rt* and *tw* mutant flies. We first examined age-related changes in climbing activities. In *rt* mutants, which showed specific reduction of *rt* transcripts (Fig. S1), the climbing abilities of flies homozygous for *rt* were significantly decreased compared to those of flies heterozygous for *rt* at all ages (Fig. 1A, Table 1). In *tw* mutants, the climbing abilities of flies homozygous for *tw* were also significantly decreased compared to those of flies heterozygous for *tw* at all ages, except at the age of 41 days between *Df(1)*/+ and *Df(1)*/*tw* (Fig. 1B, Table 2). These data showed reduced climbing abilities in *rt* and *tw* mutants at almost all ages.

**Table 1:** Statistical analysis of results of the climbing abilities of *rt* mutants.

		Days after eclosion							
Control group	Experimental group	1	6	11	16	21	26	31	36
*rt^KG^*/+	*rt^EP^*/*rt^KG^*	***	***	***	**	*	**	**	*
*rt^EP^*/+	*rt^EP^*/*rt^KG^*	***	***	***	***	***	***	***	**
*rt^KG^*/+	*rt^EP^*/+	ns	ns	ns	ns	*	*	ns	ns

*p<0.05;

**p<0.01;

***p<0.001 by Tukey test. ns, not significant.

**Table 2:** Statistical analysis of results of the climbing abilities of *tw* mutants.

		Days after eclosion										
Control group	Experimental group	1	6	11	16	21	26	31	36	41	46	51
*tw*/+	*tw*/*tw*	***	***	***	***	***	***	***	***	***	***	***
*tw*/+	*tw*/*Df(1)*	***	***	***	***	***	***	***	***	***	***	***
*Df(1)*/+	*tw*/*Df(1)*	***	***	***	***	***	**	*	*	ns	***	***
*tw*/+	*Df(1)*/+	ns	*	ns	*	ns	***	***	***	***	*	***

*p<0.05;

**p<0.01;

***p<0.001 by Tukey test. ns, not significant.

To understand the response of these mutants to milder exercises, we also evaluated the general locomotive activities of flies. The number of times a fly crossed the center of the glass tube was in the range of approximately 500–1000 in the *rt* group at all ages and was almost equal (Fig. 1C). Likewise, in the *tw* group, the number of times a fly crossed the center was in the range of approximately 1000–1800 at all ages, and we could not find any differences in the numbers between the *tw* mutant and the wild-type fly (Fig. 1D). These data showed that locomotive activities in *rt* and *tw* mutants did not decrease compared to those in wild-type flies.

Climbing ability and locomotive activities reflect leg muscle function. In order to determine the status of another behavioral function involved in other muscles, we evaluated flying ability, which reflects the function of flight muscles in the thorax as well as that of the relevant nervous system. The flying index of *rt^EP^*/*rt^KG^* flies was significantly lower than that of *rt^EP^*/+ and *rt^KG^*/+ flies (*p*<0.001, Tukey test) (Fig. 1E). In the case of *tw* mutants, the flying index of *tw*/*tw* flies was approximately half that of *tw/+* flies, and the flying index of *tw*/*Df(1)* flies was approximately three-quarters that of *Df(1)*/+ flies. There were significant differences between *tw*/*tw* and *tw/+*, and between *tw*/*Df(1)* and *tw/+* or *Df(1)*/+ (each *p*<0.001, Tukey test) (Fig. 1F). The above results showed that flying ability was reduced in *rt* and *tw* mutants.

We further examined whether defects in the flying ability of *tw* mutants were rescued by overexpression of the *tw* gene. The flying index of [*tw*/*tw*; *Act5*-*Gal4*/+; *UAS*-*tw*/+] (rescued *tw* mutant) flies increased compared to that of [*tw*/*tw*; *Act5C*-*Gal4*/+; +/+ or *tw*/*tw*; +/+; *UAS*-*tw*/+] flies (*tw* mutant) (each *p*<0.001, Tukey test) and did not differ from that of [+/+; *Act5C*-*Gal4*/+; +/+] or [+/+; +/+; *UAS*-*tw*/+] control flies (not significant, Tukey test) (Fig. 1G). These data showed that flying ability was completely rescued in *tw* mutants by overexpression of the *tw* gene. This clearly demonstrates that the *tw* gene plays an important role in motor function.

Here, we showed 3 behavioral features of *rt* and *tw* mutants: (1) mutant flies have impaired motor function; (2) defective motor function is observed from just after eclosion to death; and (3) mutant flies show abnormalities in heavy exercises such as climbing or flying, but not in light movements such as locomotion. These results were compatible with the idea that these mutant flies had abnormal muscles and/or motor neurons.

### Behavioral defects of flies expressing RNAi for the *rt* gene

**Figure 2:**
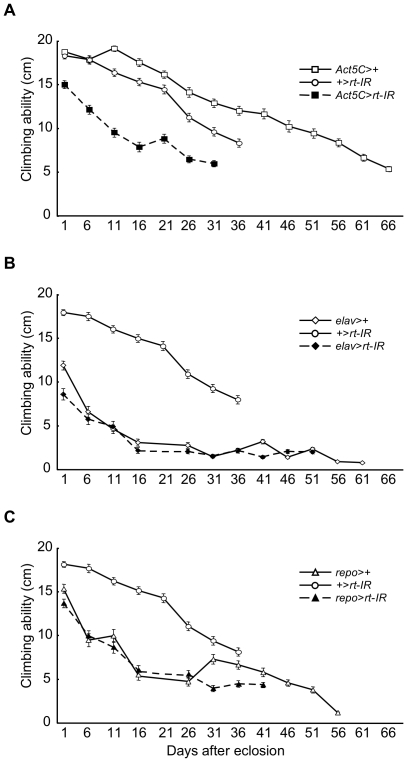
Climbing ability in flies expressing RNAi for the *rt* gene. The climbing ability of whole-body knockdown flies using *Act5C*-*Gal4* (A), that of neuron-specific knockdown flies using *elav*-*Gal4* (B), and that of glial cell-specific knockdown flies using *repo*-*Gal4* (C). Error bars indicate standard error. Results of the statistical analyses in (A), (B), and (C) are shown in Table 3. The climbing ability of flies expressing RNAi for the *rt* gene driven by *Act5C*-*Gal4* was significantly less than that of control flies at all ages. Neither neuron- nor glial cell-specific knockdown of the *rt* gene resulted in a distinct reduction in climbing ability at any age except at 1 day after eclosion in neuron-specific knockdown flies.

We examined in which tissue the expression of the *rt* and *tw* genes affects motor function. We used tissue-specific knockdown flies produced by the Gal4-UAS-IR technique [32], the tissue-specific gene knockdown technique that uses the Gal4-UAS system and RNA interference (RNAi) methods. Tissue-specific *rt* gene knockdown was induced when flies had both Gal4 driver and *UAS*-*rt*-*IR*, whereas gene knockdown was not induced when flies had only Gal4 driver or *UAS*-*rt*-*IR* (Fig. S2). The climbing ability of flies with ubiquitous expression of RNAi for the *rt* gene as driven by *Act5C*-*Gal4* was significantly lower than that of control flies at all ages (Fig. 2A, Table 3). We also examined the climbing ability of neuron- and glial cell-specific knockdown flies by using *elav-Gal4* and *repo-Gal4*, respectively, since it has been reported that there are some defects in the efficacy of synaptic transmission and changes in the subunit composition of postsynaptic glutamate receptors at the larval neuromuscular junction of *rt* mutants [24]. Neither neuron- nor glia-specific knockdown of the *rt* gene resulted in a distinct reduction in climbing ability at any age, except at the age of 1 day after eclosion in neuron-specific knockdown flies (Figs. 2B and C, Table 3). It was noted that the presence of *elav*-*Gal4* or *repo*-*Gal4* has a deteriorative effect on climbing ability for unknown reasons. These data indicated that the climbing ability of adult flies is not mainly influenced by knockdown of the *rt* gene in neurons or glial cells, and that the expression of this gene in muscles should be relevant to climbing ability.

**Table 3:** Statistical analysis of results of the climbing abilities of flies expressing RNAi for the *rt* gene.

		Days after eclosion							
Control group	Experimental group	1	6	11	16	21	26	31	36
*Act5C-Gal4*/+	*Act5C-Gal4*/*UAS*-*rt*-*IR*	***	***	***	***	***	***	***	
+/*UAS*-*rt*-*IR*	*Act5C-Gal4*/*UAS*-*rt*-*IR*	***	***	***	***	***	***	***	
*Act5C-Gal4*/+	+/*UAS*-*rt*-*IR*	ns	ns	**	*	ns	**	***	
*elav*-*Gal4*/+; +/+	*elav*-*Gal4*/+; +/*UAS*-*rt*-*IR*	**	ns	ns	ns		ns	ns	ns
+/+; +/*UAS*-*rt*-*IR*	*elav*-*Gal4*/+; +/*UAS*-*rt*-*IR*	***	***	***	***		***	***	***
*elav*-*Gal4*/+; +/+	+/+; +/*UAS*-*rt*-*IR*	***	***	***	***		***	***	***
+/+; +/*repo*-*Gal4*	+/*UAS*-*rt*-*IR*; *repo*-*Gal4*/+	ns	ns	ns	ns		ns	***	*
+/*UAS*-*rt*-*IR*; +/+	+/*UAS*-*rt*-*IR*; *repo*-*Gal4*/+	***	***	***	***		***	***	***
+/+; +/*repo*-*Gal4*	+/*UAS*-*rt*-*IR*; +/+	**	***	***	***		***	*	ns

*p<0.05;

**p<0.01;

***p<0.001 by Tukey test. ns, not significant.

### Age-related abnormal patterning and ultrastructural abnormalities in muscles of *rt* and *tw* mutants

**Figure 3:**
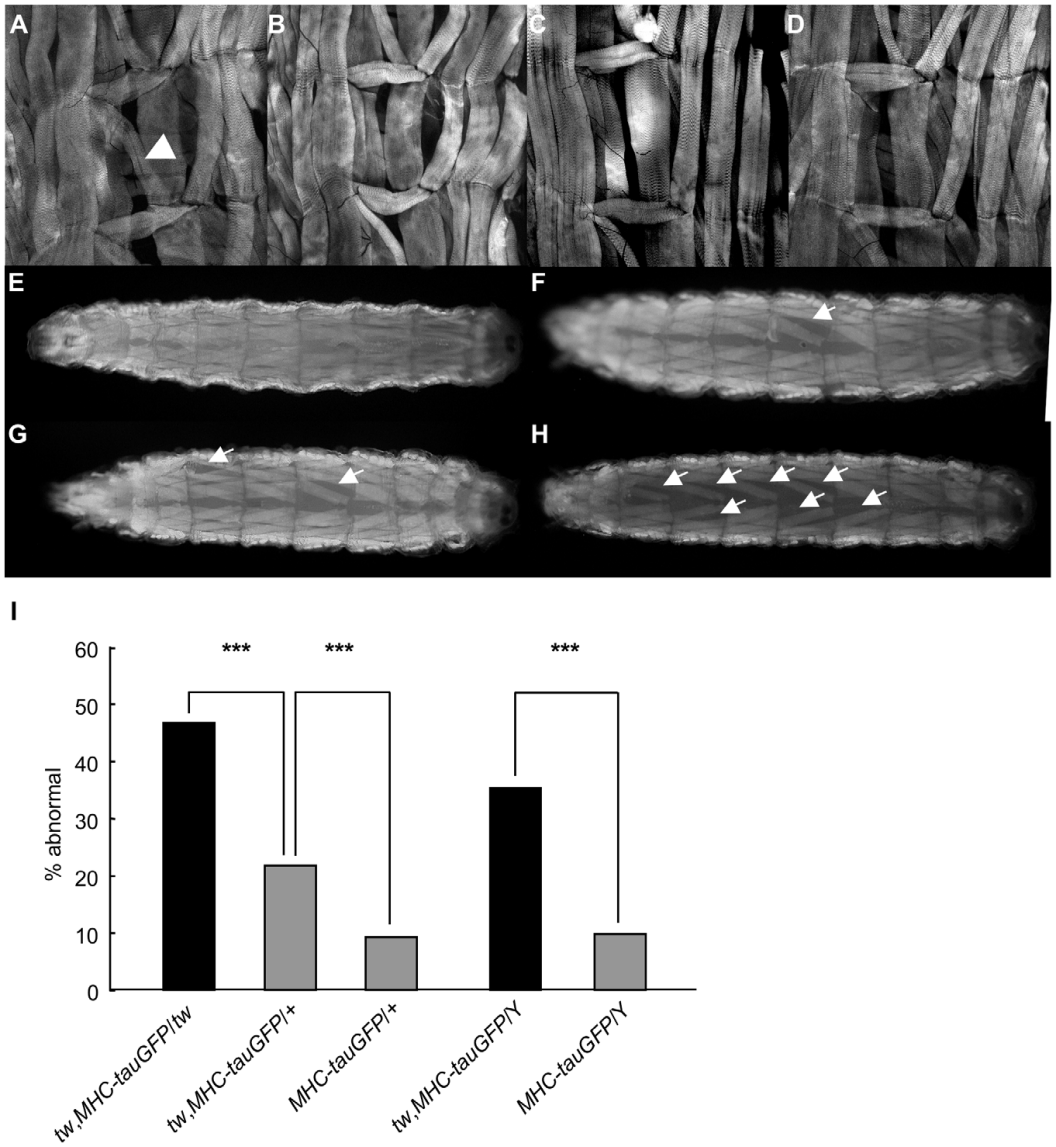
Larval body wall muscles in *rt* and *tw* mutant flies. Larval body wall muscles in abdominal segments 2–4 of wild-type flies (A), *rt^EP^*/*rt^KG^* flies (B), *rt^2^*/*rt^2^* flies (C), and flies expressing RNAi for the *tw* gene (*Act5C*>*tw*-*IR*) (D). These muscles were stained by FITC-phalloidin. The arrowhead in (A) shows muscle 5. Deficient or thin muscle 5 were observed in *rt* mutants and in flies with ubiquitous expression of RNAi for the *tw* gene. We visualized live muscles by using a *MHC*-*tauGFP* marker in wild-type (*MHC*-*tauGFP*/Y) (E) and *tw* mutant (F–H) (*tw*, *MHC*-*tauGFP*/Y) larvae. The arrows indicate the absent muscles. A few muscles were absent in the mutant larvae (F and G), and many muscles were absent in rare cases of mutant larvae (H). The frequencies of abnormal patterning of larval body wall muscles in *tw* mutants (I). Error bars indicate standard error. The frequencies of abnormal patterning of the muscles were significantly higher in *tw* mutants. ***p<0.001 by Fisher's exact test.

The behavioral data strongly suggest that *Drosophila POMT* mutants exhibit the muscle defect. Muscles of WWS patients also show structural abnormalities. Hence, we examined the effect of *rt* or *tw* on the patterning of muscles by checking the larval body wall muscles in mutants and in flies expressing RNAi for the *rt* gene. The normal structure of wild-type muscles is shown in Fig. 3A. Deficient or thin muscles were observed in the abdominal segment of *rt* mutants (Figs. 3B and C) and in flies expressing RNAi for the *tw* gene (Fig. 3D). The frequency of abnormal patterning of muscles detected by FITC-phalloidin staining in these mutants was approximately 10% (data not shown). In addition, we examined the frequency of abnormal patterning in larval muscles of *tw* mutants by using the *MHC-tauGFP* marker to observe live muscles. In *MHC-tauGFP* larvae, which did not show reduction of *rt* and *tw* transcripts (Fig. S3), the dorsal body wall muscles were structurally normal (Fig. 3E). A few muscles were absent in the mutant larvae (Figs. 3F and G), and many muscles were absent in rare cases of mutant larvae (Fig. 3H). Significantly higher frequencies of abnormal patterning were detected in the muscles of *tw* mutants (probabilities for all compared pairs were *p*<0.001, Fisher's exact test) (Fig. 3I).

**Figure 4:**
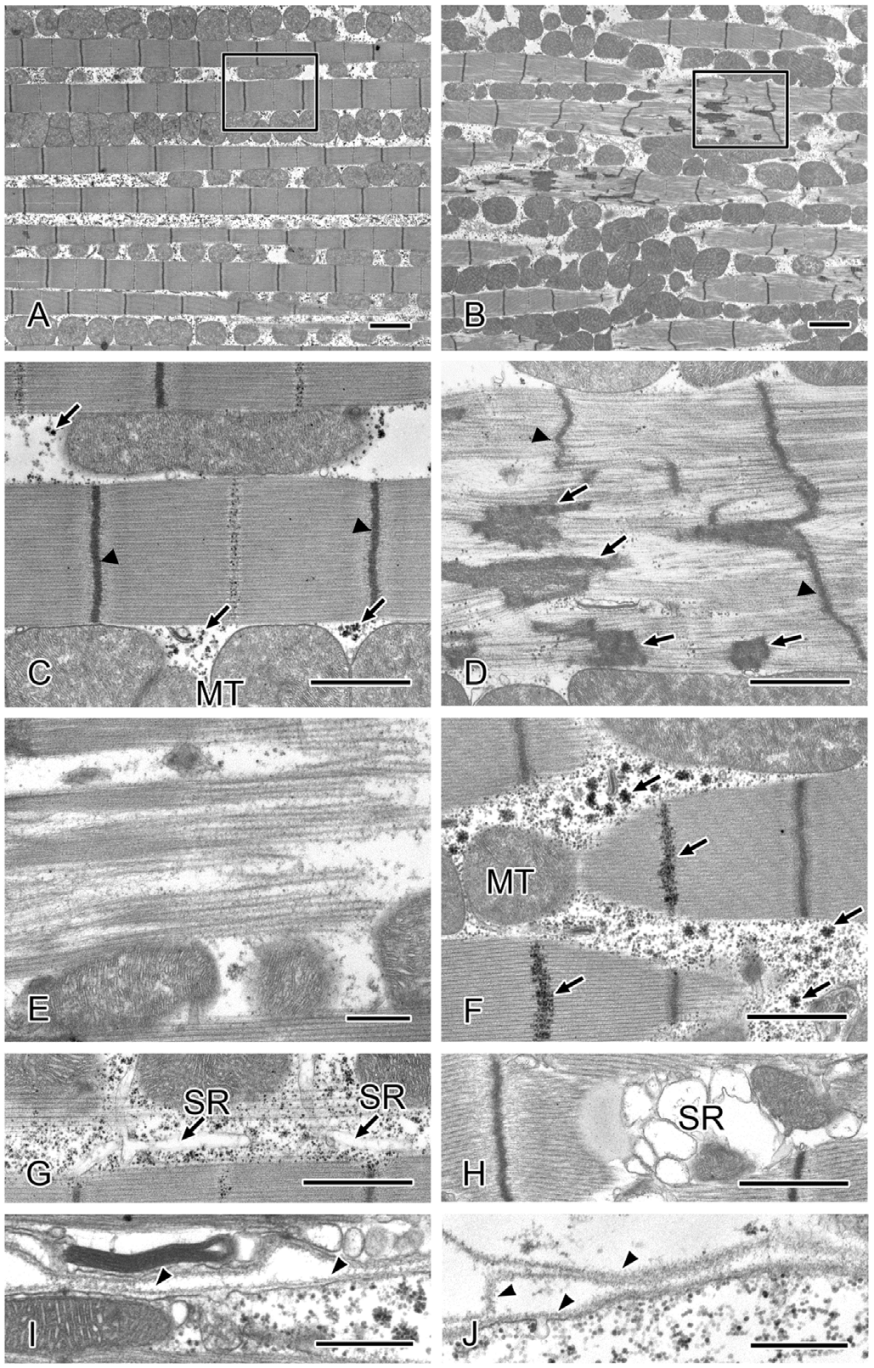
Representative electron micrographs of thoracic muscles in aged wild-type and *tw* mutant flies. (A, C, G, and I) Thirty-five-day-old wild-type fly muscles. (B, D, E, F, H, and J) Thirty-five-day-old *tw* mutant fly muscles. (A and B) Low-magnification images of muscles. (C and D) High-magnification view of the area bordered by the rectangle in Figs. 4A and B. (C) Normal sarcomere with regular Z-lines (arrowheads). (D) Z-lines (arrowheads) are irregular and often streaming. Nemaline bodies (arrows) in the muscle fiber. (E) Actin and myosin filaments are disorganized. (F) Glycogen granules (arrows) are accumulated. (G) Normal sarcoplasmic reticulum (SR). (H) SR is swollen. (I) Normal basement membrane. (J) The basement membrane (arrowheads) is duplicated and multilayered. MT: mitochondria. Bars: (A and B) 2 µm, (C, D, F, G, and H) 1 µm, and (E, I, and J) 500 nm.

**Figure 5:**
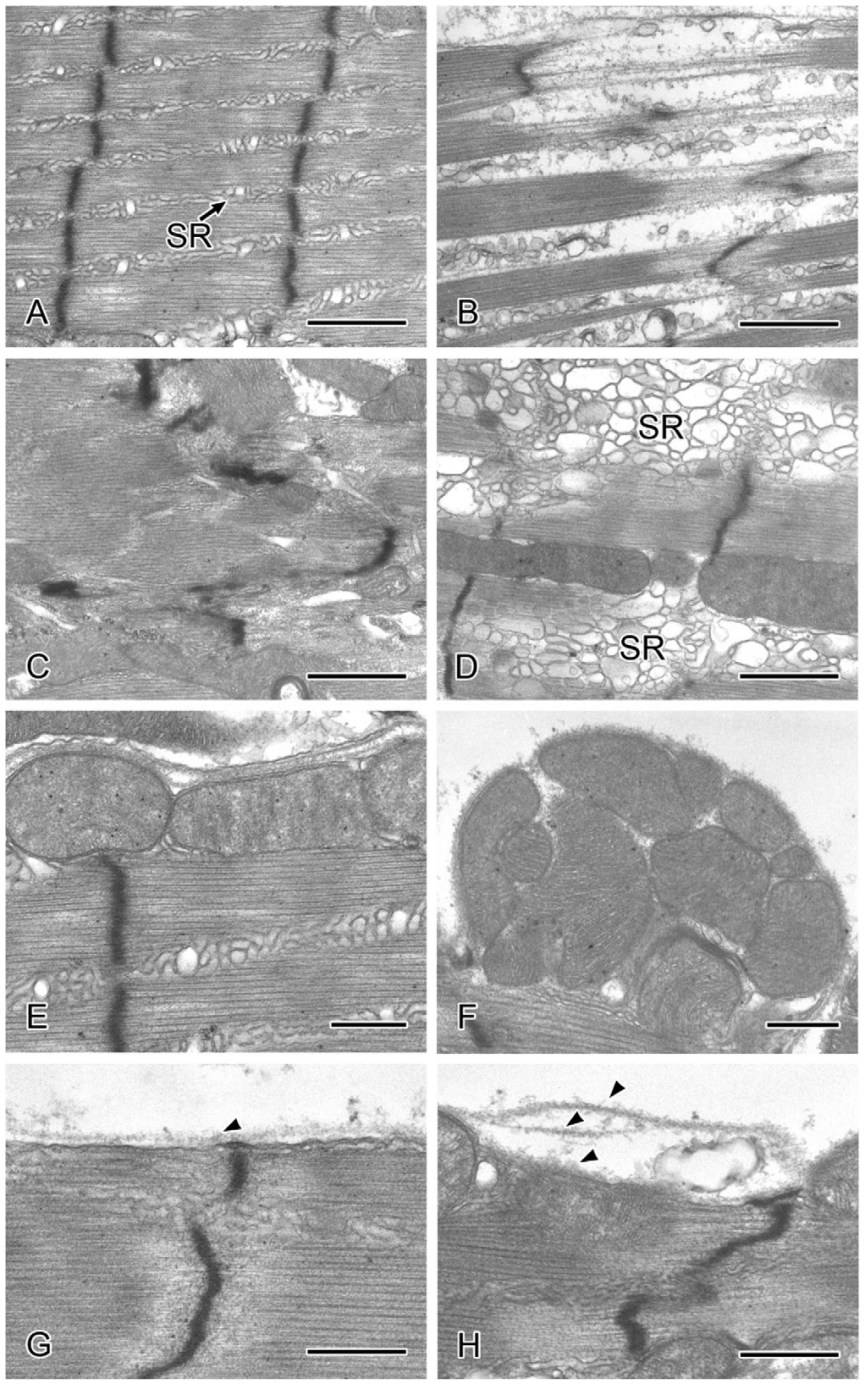
Representative electron micrographs of leg muscles in aged wild-type and *tw* mutant flies. (A, E, and G) Muscles of 35-day-old wild-type flies. (B, C, D, F, and H) Muscles of 35-day-old *tw* mutant flies. (A) Normal sarcomere in a wild-type fly with regular Z-lines. (B) In the *tw* mutant, numbers of actin and myosin filaments are decreased and disorganized. (C) Z-lines are irregular and often incomplete. (D) Sarcoplasmic reticulum is swollen. Normal mitochondria are observed in wild-type fly muscles (E) while enlarged mitochondria are accumulated in *tw* mutant fly muscles (F). The normal basement membrane (arrowhead) runs continuously along the sarcolemma in wild-type fly muscles (G), while the basement membrane (arrowheads) is duplicated and multilayered in the *tw* mutant fly muscles (H). SR: sarcoplasmic reticulum. Bars: (A–D) 1 µm and (E–H) 500 nm.

**Figure 6:**
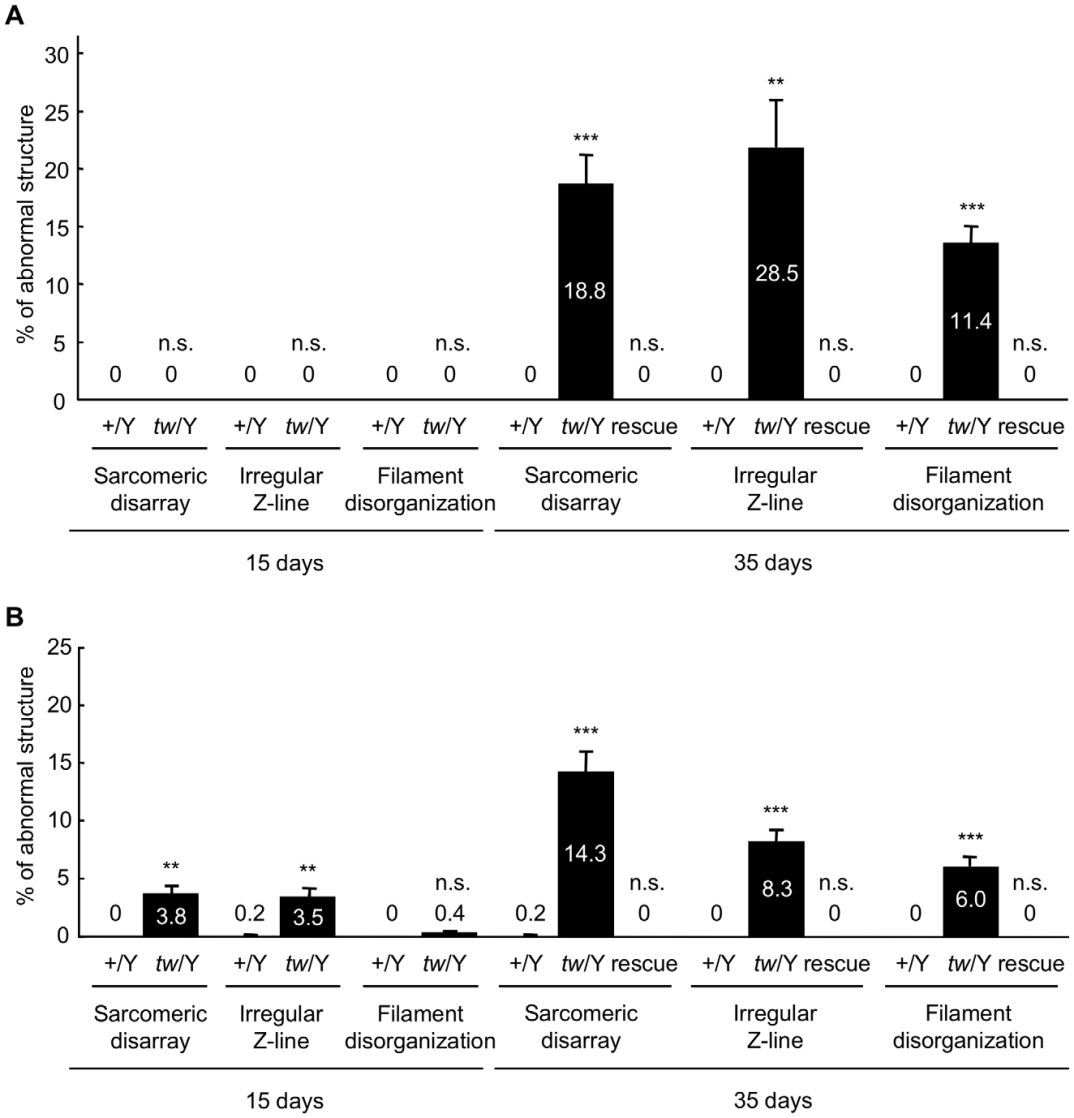
Frequency of abnormal structures in the muscles of *tw* mutant flies. (A) Thoracic muscles. (B) Leg muscles. The percentages of sarcomeric disarray, irregular Z-line, and filament disorganization noted in the muscle area of 590 µm^2^ per individual in 15- and 35-day-old wild-type and *tw* mutant flies are shown. In thoracic muscles, these abnormal structures were observed in 35-day-old mutant flies but not in wild-type flies. In leg muscles, these abnormal structures were observed in both 15- and 35-day-old mutant flies but were hardly detected in wild-type flies. We compared data of each abnormal phenotype in 15- and 35-day-old wild-type, *tw* mutant, and rescued flies. Each bar represents the mean of 6 individuals. Error bars indicate standard error. **p<0.01, ***p<0.001 by Welch's two sample *t* test. n.s., not significant.

We further examined the effect of the *tw* mutation on sarcomeric structure by performing detailed electron microscopic analysis on the leg and thoracic muscles from wild-type, *tw* mutant and the rescue flies. Similar changes in ultrastructure were observed in the leg and thoracic muscles of both male and female *tw* mutant flies (Table 4). The normal sarcomeric structure of the muscles of wild-type flies is shown in Figs. 4A, 4C, and 5A. Sarcomeric disarray was frequently observed in the muscles of *tw* mutant flies (Figs. 4B, 4D, 5B, and 5C). In the mutant muscles, Z-lines were irregular and often streaming (Figs. 4D, 5B, and 5C), nemaline bodies were observed in the muscle fiber (Fig. 4D), actin and myosin filaments were disorganized (Figs. 4E and 5B), and accumulated glycogen granules were seen (Fig. 4F). Enlarged mitochondria (Fig. 5F) and swollen sarcoplasmic reticulum (SR) were seen in the *tw* mutant muscles (Figs. 4H and 5D), while normal mitochondria and SR were observed between the muscle fibers (Figs. 4A, 4C, 4G, 5A, and 5E). The basement membrane was duplicated and multilayered in *tw* mutant muscles (Figs. 4J and 5H), while normal basement membrane was observed continuously along the sarcolemma in wild-type muscles (Figs. 4I and 5G). The abovementioned defective muscle phenotypes were observed both in 15- and 35-day-old *tw* mutants but were hardly detected in wild-type flies. The number of mutants with defective phenotypes was higher in 35-day-old *tw* mutants than in 15-day-old *tw* mutants. Moreover, these defective phenotypes could not be found among the rescue flies (Table 4), indicating that the defective phenotypes in the mutants were fully rescued. These results demonstrated that *tw* contributed to the maintenance of muscle ultrastructure. Next, we counted the number of sarcomeric disarray, irregular Z-line, and filament disorganization occurrences in a 590-µm^2^ muscle area per individual and calculated the percentage of these abnormal structures (number of abnormal structures/number of observed). In thoracic muscles, these abnormal structures were observed in 35-day-old mutants but could not be detected in wild-type flies (Fig. 6A). In leg muscles, these abnormal structures were observed in both 15- and 35-day-old mutants but were hardly detected in wild-type flies (Fig. 6B). The percentages of abnormal structures in the *tw* mutant were significantly higher than those in wild-type flies except for filament disorganization in the leg muscles of 15-day-old flies, when abnormal structures were observed (Fig. 6). Moreover, these abnormal structures were significantly exaggerated with age in *tw* mutants but not significantly exaggerated with age in wild-type flies except for sarcomeric disarray in the leg muscles of 15-day-old flies (Table 5). These changes, which are reminiscent of the progressive symptoms in WWS patients, were more frequently observed in 35-day aged mutant muscles than in 15-day aged mutant muscles. The abovementioned results clearly demonstrated several kinds of abnormalities in the muscles of *tw* mutants that become more severe with age.

**Table 4:** Ultrastructure defects of thoracic and leg muscles from 15- and 35-day-old *tw* mutants.

	15-day-old fly muscles				35-day-old fly muscles					
	Thorax		Leg		Thorax			Leg		
Abnormal phenotype	+/Y	*tw*/Y	+/Y	*tw*/Y	+/Y	*tw*/Y	rescue	+/Y	*tw*/Y	rescue
sarcomeric disarray *	0/6	0/6	0/6	6/6	0/6	6/6	0/6	1/6	6/6	0/6
irregular Z-lines *	0/6	0/6	2/6	6/6	0/6	6/6	0/6	0/6	6/6	0/6
filament disorganization *	0/6	0/6	0/6	1/6	0/6	6/6	0/6	0/6	6/6	0/6
swollen SR	0/6	6/6	0/6	6/6	0/6	6/6	0/6	0/6	6/6	0/6
accumulation of glycogen	0/6	0/6	0/6	0/6	0/6	6/6	0/6	0/6	6/6	0/6
mitochondrial enlargement	0/6	6/6	0/6	6/6	0/6	6/6	0/6	0/6	6/6	0/6
basement membrane dup.	0/6	0/6	0/6	0/6	0/6	6/6	0/6	0/6	6/6	0/6

(Number of individuals that have abnormal phenotype)/(Number of individuals observed) is shown. The “rescue” genotype was [*tw*/Y; *Act5C-Gal4*/+; *UAS-tw*/+]. A muscle area of 590 µm^2^ per individual was observed.

*: The number of abnormal phenotypes in the muscle area of 590 µm^2^ per individual was counted and the percentage of abnormal structures (Number of abnormal structures/Number of structures observed) was calculated and is shown in Figure 6. SR, sarcoplasmic reticulum.

**Table 5:** Numbers of abnormal muscle ultrastructures increasing with age in *tw* mutant flies.

Muscle	Abnormal phenotype	+/Y	*tw*/Y
Thorax	sarcomeric disarray	ns	***
	irregular Z-lines	ns	**
	filament disorganization	ns	***
Leg	sarcomeric disarray	*	**
	irregular Z-lines	ns	**
	filament disorganization	ns	***

The percentage of abnormalities observed in 35-day-old flies compared to that in 15-day-old flies of the same genotype. The percentage of abnormalities was significantly exaggerated with age in *tw* mutant flies but not significantly exaggerated with age in wild-type flies except for sarcomeric disarray in the leg muscles of 15-day-old flies.

*p<0.05;

**p<0.01;

***p<0.001 by Welch's two sample *t* test. ns, not significant.

### Myoblasts in the wing discs of *tw* mutants

**Figure 7:**
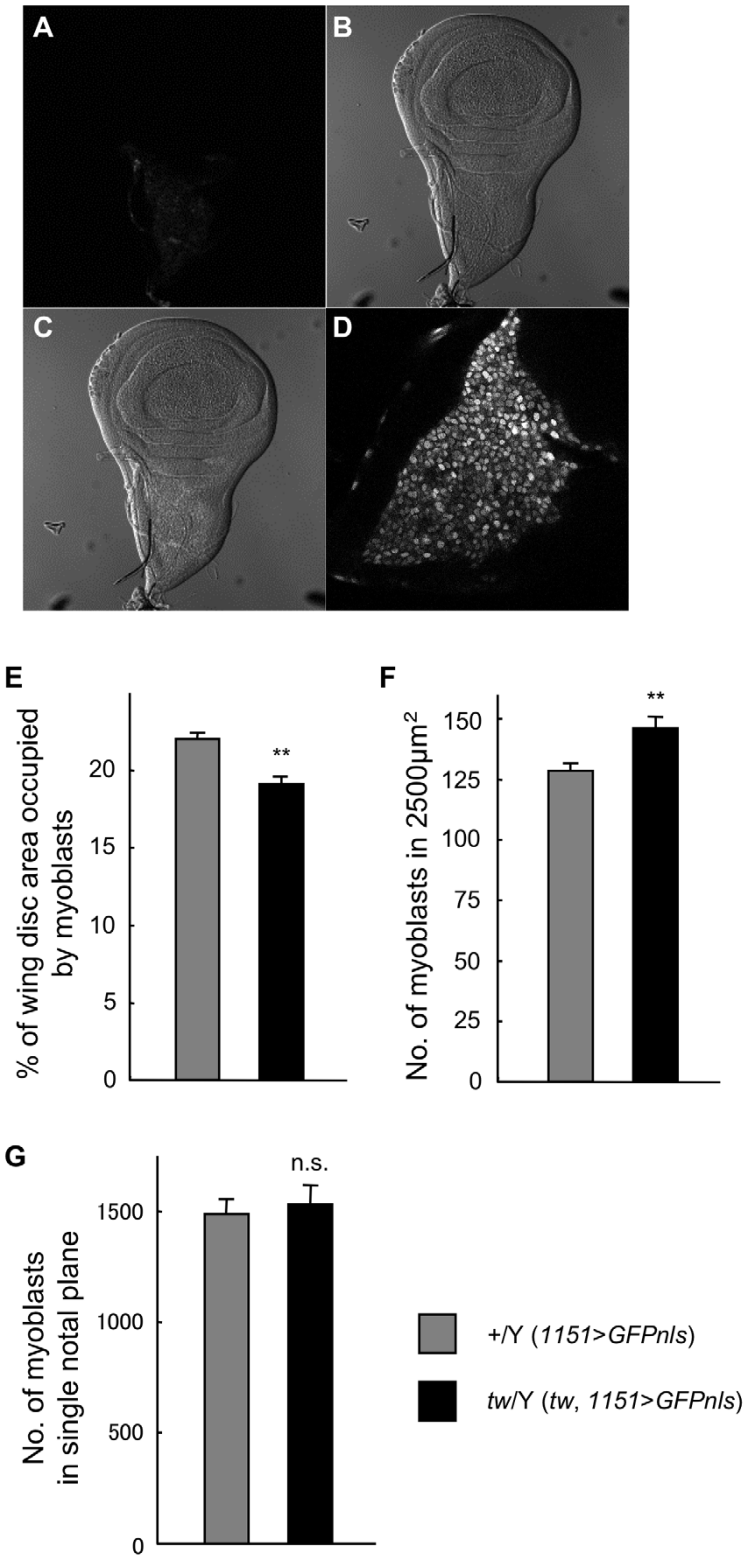
Myoblasts in the wing imaginal disc of *tw* mutant larvae. (A) Myoblasts in the wing imaginal disc of wild-type larvae. Myoblasts were visualized by nuclear localization of GFP (GFPnls), which was driven by *1151*-*Gal4*. (B) Wing imaginal disc of wild-type (*1151*>*GFPnls*) larvae. (C) Merged image of (A) and (B). (D) High-magnification image of the notum plane region in the wing disc. Myoblast nuclei are seen as white or gray spots. (E) The percentage of wing imaginal disc area occupied by myoblasts in wild-type and *tw* mutant (*tw*, *1151*>*GFPnls*) larvae. (F) The number of myoblasts in a 2500 µm^2^ area of the wing imaginal disc in wild-type and *tw* mutant larvae. (G) Total number of myoblasts in a single notum plane region in the wing imaginal disc of wild-type and *tw* mutant larvae. Error bars in E–G indicate standard error. The wing disc area occupied by myoblasts in *tw* mutant flies was less than that in the wild-type flies. The number of myoblasts in the constant area of the mutant fly was more than that of the wild-type fly. The total number of myoblasts in a single wing disc was equal in the *tw* mutant and wild-type flies. The density of myoblasts in *tw* mutant larvae was higher than that in wild-type larvae. **p<0.01 by *t* test. n.s., not significant.

In *Drosophila*, flight muscles in the thorax develop from myoblasts in the wing imaginal discs of larvae. The climbing ability of the *tw* mutant at 1 day of age was less than that of the wild-type fly (Fig. 1B). These facts suggest that something happens to the myoblasts of *tw* mutants. We determined whether the number of myoblasts in the *tw* mutant changed by observing myoblasts in third instar larvae. Myoblasts were present in the nodal plane of the third instar larva (Figs. 7A–D). The wing disc area occupied by myoblasts in [*tw*, *1151*-*Gal4*/Y; *UAS*-*GFPnls*/+] (*tw* mutant) flies was less than that in [*1151*-*Gal4*/Y; *UAS*-*GFPnls*/+] (wild-type) flies (Fig. 7E). However, the number of myoblasts per unit area of mutant tissue was higher than that of wild-type tissue (Fig. 7F). As a result, the total number of myoblasts in a single wing disc was approximately 1500, which was nearly equal in *tw* mutants and wild-type flies (Fig. 7G). These data showed that the density of myoblasts in the *tw* mutant was higher than that in the wild-type fly.

### Excessive apoptosis of myoblasts in the wing disc of the *tw* mutant

**Figure 8:**
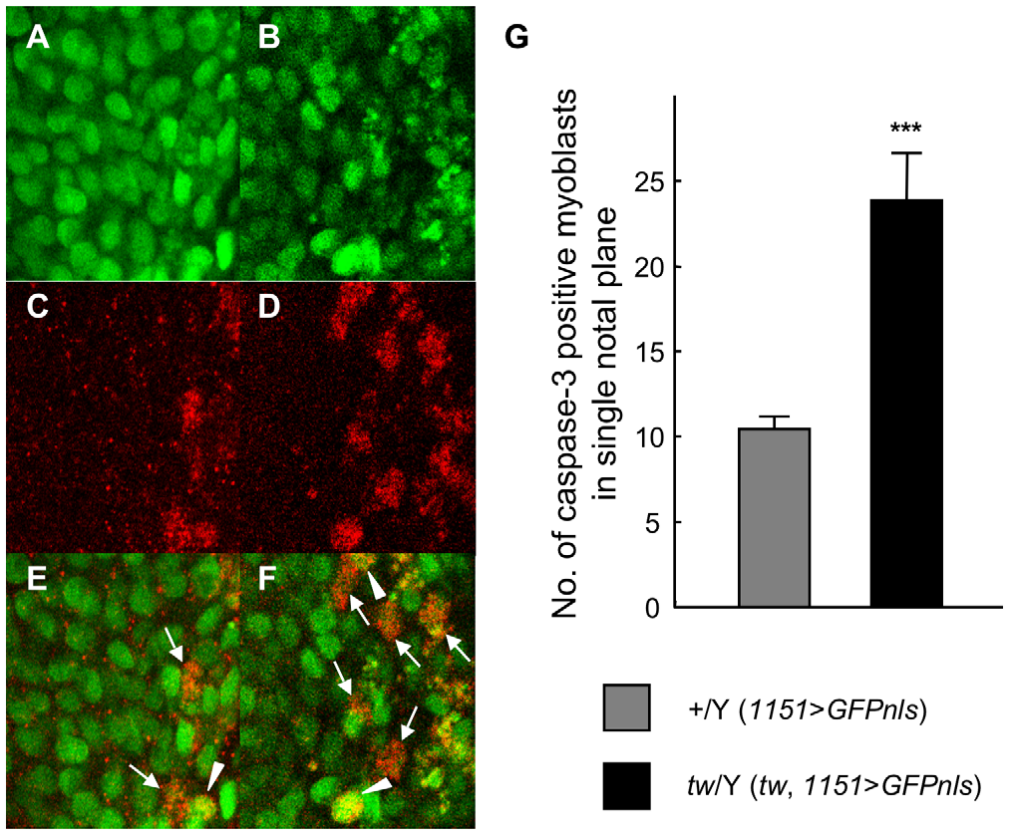
Excessive apoptosis of myoblasts in the wing imaginal disc of *tw* mutant larva. (A, C, and E) The wild-type fly (*1151*>*GFPnls*). (B, D, and F) The *tw* mutant fly (*tw*, *1151*>*GFPnls*). (A) and (B) Myoblasts in the wing imaginal disc of larvae. The GFP localize in myoblast nuclei. (C) and (D) Myoblasts stained by caspase-3 antibody, a marker of apoptotic cells. (E) and (F) Merged images of (A) and (C) and of (B) and (D), respectively. The arrowheads and arrows show the co-localization of GFP and caspase-3. The arrowheads show the nuclei just before breakdown. The arrows show degraded nuclei in more of a progressive apoptotic stage than the nuclei shown by arrowheads. (G) The number of myoblasts positive for caspase-3 in the wing imaginal disc of wild-type and *tw* mutant larvae. Error bars indicate standard error. The number of apoptotic myoblasts in *tw* mutant larvae was significantly lower than that in wild-type larvae. ***p<0.001 by *t* test.

Changing of the density of myoblasts is a result of an alteration in cell death or cell division. Therefore, we checked the number of apoptotic and dividing myoblasts. The nuclei of myoblasts were visualized by GFP (Figs. 8A and B), and we observed the apoptotic myoblasts by using the cleaved caspase-3 antibody (Figs. 8C and D). Both wild-type flies and *tw* mutants had apoptotic myoblasts (Figs. 8E and F); however, the number of apoptotic myoblasts in the wing discs of *tw* mutants was 2.4-fold higher than that in the wing discs of wild-type flies (Fig. 8G). We also observed dividing myoblasts by using phospho-histone H3 antibody. The number of dividing myoblasts in the wing disc did not differ between wild-type flies and *tw* mutants (Fig. S4). These results showed that apoptosis was enhanced in myoblasts of the *tw* mutant while the number of dividing cells was not altered. Excessive apoptosis of myoblasts during muscle differentiation should lead to muscle disorganization, including muscle cell defects.

### Increased α-spectrin in myoblasts and βPS-integrin around myoblasts of the *tw* mutant

**Figure 9:**
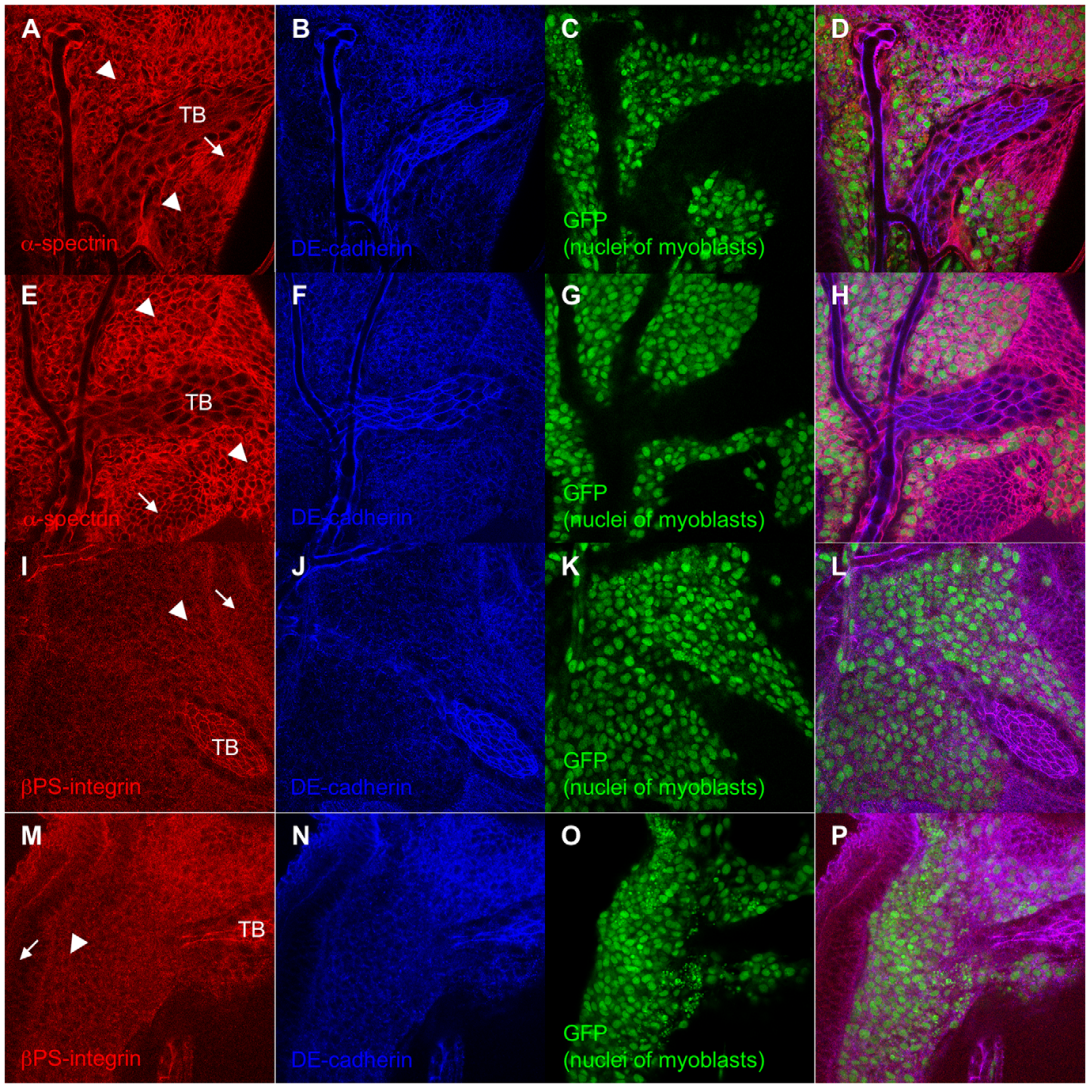
Increased α-spectrin and βPS-integrin in the myoblasts of *tw* mutant. (A–D and I–L) wild type (*1151*>*GFPnls*). (E–H and M–P) *tw* mutant (*tw*, *1151*>*GFPnls*). (A) and (E) Images stained by anti-α-spectrin antibody. α-spectrin is a component of cytoskeleton inside of cell membrane and bind to actin. (B), (F), (J), and (N) Images stained by anti-DE-cadherin antibody. DE-cadherin is a cell adhesion molecule located in cell surface. (C), (G), (K), and (O) Nuclei of myoblasts. (D), (H), (L), and (P) Merged images of (A–C), (E–G), (I–K), and (M–O), respectively. (I) and (M) Images stained by anti-βPS-integrin antibody. βPS-integrin is a cell adhesion molecule located in cell surface and bind to extracellular matrix. TB, arrowheads, and arrows in (A), (E), (I), and (M) show tracheoblast, the region of myoblasts, and lateral region of myoblasts, respectively. The signals of α-spectrin and βPS-integrin excessively increased in the region of myoblasts compared to the lateral region of myoblasts in *tw* mutant although apoptosis increased in the myoblasts of *tw* mutant. These signals in wild type did not change between two regions. But we could not find any difference in the signal of DE-cadherin.

Cytoskeletal and cell adhesion molecules, such as spectrins, cadherins, and integrins, proteolyze during apoptosis [33]–[35]. Thus, we examined the expression of α-spectrin, DE-cadherin, and βPS-integrin in myoblasts of the *tw* mutant. Surprisingly, the signals of α-spectrin and βPS-integrin excessively increased in the region of myoblasts compared to the lateral region of myoblasts in the *tw* mutant (Figs. 9E and M) although apoptosis increased in the myoblasts of *tw* mutant. These signals in wild-type flies did not change between the 2 regions (Figs. 9A and I). We could not find any difference in the DE-cadherin signal (Figs. 9B, F, J, and N). These data suggest that α-spectrin and βPS-integrin might be induced to protect myoblasts of the *tw* mutant from apoptosis.

### Lethality in *tw* mutants and flies expressing RNAi for the *rt* gene

**Figure 10:**
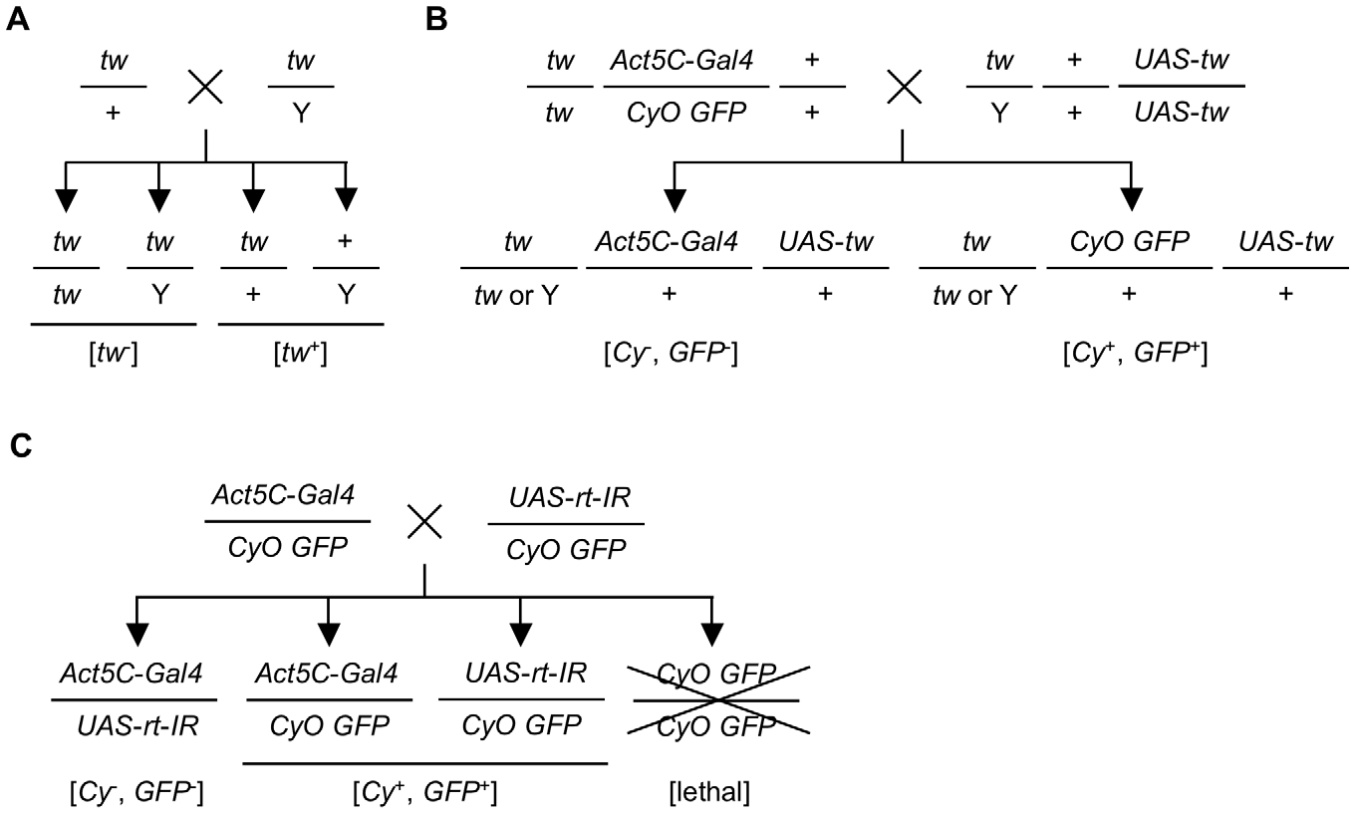
Crosses for examining *tw* and *rt* mutant fly viability. (A) The cross scheme for examining *tw* mutant fly viability. Female *tw*/+ flies were crossed with male *tw* mutants (*tw*/Y). Genotypes of F_1_ progeny are *tw*/*tw*, *tw*/Y, *tw*/+, and +/Y. Individuals with genotype *tw*/*tw* or *tw*/Y have the twisted abdominal phenotype, which is represented as [*tw* ^−^]. On the other hand, individuals with genotype *tw*/+ or +/Y have the normal abdominal phenotype, which is represented as [*tw* ^+^]. The number of F_1_ progeny with phenotype [*tw* ^−^] or [*tw* ^+^] is shown in Table 6. (B) The cross scheme for the rescue experiment of the *tw* mutant. Female *Act5C*-*Gal4* driver flies with *tw* mutation were crossed with male *UAS*-*tw* flies with *tw* mutation. In the F_1_ progeny, rescued and non-rescued individuals are born and are described as [*Cy* ^−^, *GFP* ^−^] and [*Cy* ^+^, *GFP* ^+^], respectively. The number of F_1_ progeny with phenotypes [*Cy* ^−^, *GFP* ^−^] or [*Cy* ^+^, *GFP* ^+^] is shown in Table 7. (C) The cross scheme for examining the viability of flies expressing RNAi for the *rt* gene. Female *Act5C*-*Gal4* driver flies were crossed with male *UAS*-*rt*-*IR* flies. In the F_1_ progeny, flies expressing RNAi for the *rt* gene, *Act5C*-*Gal4* driver, and *UAS*-*rt*-*IR* individuals were born. Flies expressing RNAi for the *rt* gene do not have *CyO GFP* balancer; thus, the phenotype is described as [*Cy* ^−^, *GFP* ^−^]. *Act5C*-*Gal4* driver and *UAS*-*rt*-*IR* flies have *CyO GFP* balancer; thus, the phenotype is described as [*Cy* ^+^, *GFP* ^+^]. The number of F_1_ progeny with ([*Cy* ^−^, *GFP* ^−^]) or without ([*Cy* ^+^, *GFP* ^+^]) ubiquitous expression of RNAi for the *rt* gene is shown in Table 8.

It is known that WWS has high lethality rates during early development. Therefore, we determined whether *Drosophila POMT* genes play an important role in viability. We crossed females heterozygous for *tw* with male hemizygous for *tw* and checked the number of F_1_ progenies (Fig. 10A). If all the eggs of F_1_ progenies hatched and developed normally, the ratio of the number of individuals having a twisted abdomen, the *tw* phenotype, to those having a normal abdomen is expected to be 1. The ratio was found to be 0.2, significantly lower than the expected ratio (*p*<0.001, chi-square test) (Table 6). In addition, in order to determine the necessity of the *tw* gene for viability, we performed a rescue experiment by expressing the *tw* gene in *tw* mutants (Fig. 10B). The ratio was found to be 1.25 and was significantly increased (*p*<0.001, chi-square test) (Table 7). These data indicated that the *tw* gene plays an important role in viability and normal development. We also examined fly viability after knockdown of the *rt* gene under 3 different temperature conditions. Knockdown at high temperature is more efficient than that at low temperature because yeast transcriptional factor GAL4 binds strongly to UAS sequences. Ratios of the number of knockdown flies to the number of non-knockdown flies were 0.26, 0.12, and 0.00 at 18, 25, and 28°C, respectively (Fig. 10C, Table 8). Growing flies at a higher temperature resulted in higher lethality, indicating that the *rt* gene also contributed to normal development. The abovementioned results showed that the *tw* and *rt* genes were essential for the viability of the embryo, larva, and/or pupa. Elucidation of the reason for the lethal phenotype in these mutants could clarify the mechanism of low viability in human patients.

**Table 6:** Viability of *tw* mutant flies.

Phenotype			
[*tw* ^−^]	[*tw* ^+^]	Ratio^a^ ([*tw* ^−^]/[*tw* ^+^])	*p* (*x* ^2^)
912	3307	0.28	<0.001 (1359)

a Number of adult *tw*/*tw* and *tw*/Y flies divided by number of *tw*/+ and +/Y flies. If all flies hatch and develop normally, the ratio is expected to be 1.

**Table 7:** Viability of rescued *tw* mutant flies.

Phenotype			
[*Cy* ^−^, *GFP* ^−^]	[*Cy* ^+^, *GFP* ^+^]	Ratio^a^ ([*Cy* ^−^, *GFP* ^−^]/[*Cy* ^+^, *GFP* ^+^])	*p* (*x* ^2^)
948	757	1.25	<0.001 (21.4)

a Number of adult [*tw*/*tw*; *Act5C-Gal4*/+; *UAS-tw*/+] and [*tw*/Y; *Act5C-Gal4*/+; *UAS-tw*/+] flies divided by number of [*tw*/*tw*; *CyO GFP*/+; *UAS-tw*/+] and [*tw*/Y; *CyO GFP*/+; *UAS-tw*/+] flies. If the lethal phenotype of *tw* mutant flies is rescued, the ratio is expected to be more than 1.

**Table 8:** Effect of temperature on lethality in flies expressing RNAi for the *rt* gene.

	Phenotype		
Temperature (°C)	[*Cy* ^−^, *GFP* ^−^]	[*Cy* ^+^, *GFP* ^+^]	Ratio^a^ ([*Cy* ^−^, *GFP* ^−^]/[*Cy* ^+^, *GFP* ^+^])
18	206	781	0.26
25	182	1541	0.12
28	1	405	0.00

a Number of adult *Act5C-Gal4*/*rt*-*IR* flies divided by number of *Act5C-Gal4*/+ and +/*rt*-*IR* flies. If all flies hatch and develop normally, the ratio is expected to be 0.33.

### Shortened lifespan of flies expressing RNAi for the *rt* gene

**Figure 11:**
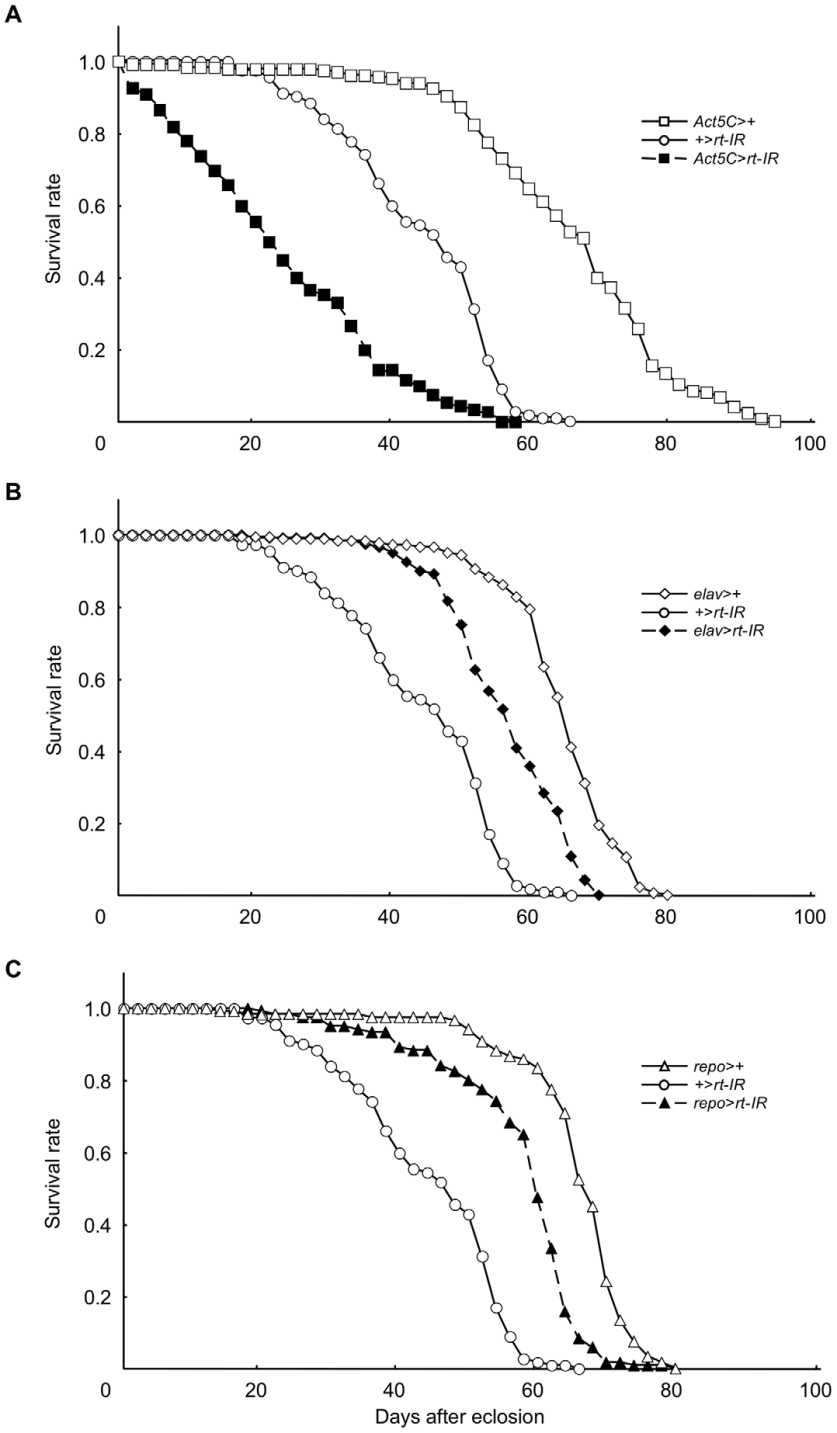
Lifespans of flies expressing RNAi for the *rt* gene. The lifespan of whole-body knockdown flies using *Act5C*-*Gal4* (A), that of neuron-specific knockdown flies using *elav*-*Gal4* (B), and that of glial cell-specific knockdown flies using *repo*-*Gal4* (C). The results of statistical analyses in (A), (B), and (C) are shown in Table 9. The median lifespan for each genotype is shown in Table 10. Flies with ubiquitous expression of RNAi for the *rt* gene driven by *Act5C*-*Gal4* (*Act5C*-*Gal4*/*rt*-*IR*) had a shorter lifespan than that of the *UAS*-*rt*-*IR*/+ and *Act5C*-*Gal4*/+ control groups (both *p*<0.001, log-rank test, Table 9). The median lifespan of *Act5C*-*Gal4*/*rt*-*IR* flies was 23 days, which was −51.1% of that of *UAS*-*rt*-*IR*/+ flies (47 days) and −66.7% of that of *Act5C*-*Gal4*/+ flies (69 days) (Tables 9 and 10). On the other hand, the lifespan of [*elav*-*Gal4*/+; *UAS*-*rt*-*IR*/+] flies and [*UAS*-*rt*-*IR*/+; *repo*-*Gal4*/+] flies was longer than that of [*UAS*-*rt*-*IR*/+; +/+] flies, one control group, and was shorter than that of [*elav*-*Gal4*/+; +/+] flies and [+/+; *repo*-*Gal4*/+] flies, the other control groups. The median lifespans of [*elav*-*Gal4*/+; *UAS*-*rt*-*IR*/+] flies and [*UAS*-*rt*-*IR*/+; *repo*-*Gal4*/+] flies were 57 and 59 days, respectively (Table 10). Both lifespans were longer than that of [*UAS*-*rt*-*IR*/+; +/+] flies and shorter than that of [*elav*-*Gal4*/+; +/+] flies and [+/+; *repo*-*Gal4*/+] flies (Table 9).

Patients with WWS rarely survive to adulthood [10]. Therefore, we investigated whether *rt* knockdown mutants had shortened lifespans. Flies with ubiquitous expression of RNAi for the *rt* gene driven by *Act5C*-*Gal4* had shorter lifespans than those of the control groups (both *p*<0.001, log-rank test, Table 9) (Fig. 11A). The median lifespan of *Act5C*-*Gal4*/*rt*-*IR* flies was 23 days, which was −51.1% of that of *UAS*-*rt*-*IR*/+ flies (47 days) and −66.7% of that of *Act5C*-*Gal4*/+ flies (69 days) (Tables 9 and 10). On the other hand, the lifespans of flies expressing RNAi for the *rt* gene in neurons and glial cells driven by *elav*-*Gal4* and *repo-Gal4*, respectively, were not affected (Figs. 11B and C, Tables 9 and 10). These results indicated that knockdown of the *rt* gene in all tissues reduced the lifespan, while knockdown in neurons or glial cells did not influence lifespan. Together with the results of the muscle phenotype in *Drosophila* POMT mutants, these results suggest that age-related weakness of the muscles in the heart and/or gastrointestinal tract may lead to early death in flies.

**Table 9:** Statistical analysis of lifespans of flies expressing RNAi for the *rt* gene.

Control group	Experimental group	% Difference in median lifespan	*p* value
+/+; +/*UAS*-*rt*-*IR*; +/+	+/+; *Act5C-Gal4*/*UAS*-*rt*-*IR*; +/+	−51.1	<0.001
+/+; *Act5C-Gal4*/+; +/+	+/+; *Act5C-Gal4*/*UAS*-*rt*-*IR*; +/+	−66.7	<0.001
+/+; +/*UAS*-*rt*-*IR*; +/+	*elav*-*Gal4*/+; +/*UAS*-*rt*-*IR*; +/+	21.3	<0.001
*elav*-*Gal4*/+; +/+; +/+	*elav*-*Gal4*/+; +/*UAS*-*rt*-*IR*; +/+	−12.3	<0.001
+/+; +/*UAS*-*rt*-*IR*; +/+	+/+; +/*UAS*-*rt*-*IR*; *repo*-*Gal4*/+	25.5	<0.001
+/+; +/*repo*-*Gal4*; +/+	+/+; +/*UAS*-*rt*-*IR*; *repo*-*Gal4*/+	−11.9	<0.001

C, control group; E, experimental group. Percent difference (calculated as [E–C]/C×100) and log-rank test *p* values are given.

**Table 10:** Median lifespan of flies expressing RNAi for the *rt* gene.

Genotype	Number of animals	Median lifespan (days)
+/+; *Act5C-Gal4*/*UAS*-*rt*-*IR*; +/+	163	23
+/+; +/*UAS*-*rt*-*IR*; +/+	112	47
+/+; *Act5C-Gal4*/+; +/+	226	69
*elav*-*Gal4*/+; +/*UAS*-*rt*-*IR*; +/+	120	57
*elav*-*Gal4*/+; +/+; +/+	180	65
+/+; +/*UAS*-*rt*-*IR*; *repo*-*Gal4*/+	120	59
+/+; +/*repo*-*Gal4*; +/+	120	67

### No enzymatic activity in the protein of the *tw* mutant form or in the larval extracts of *rt* and *tw* mutants

**Figure 12:**
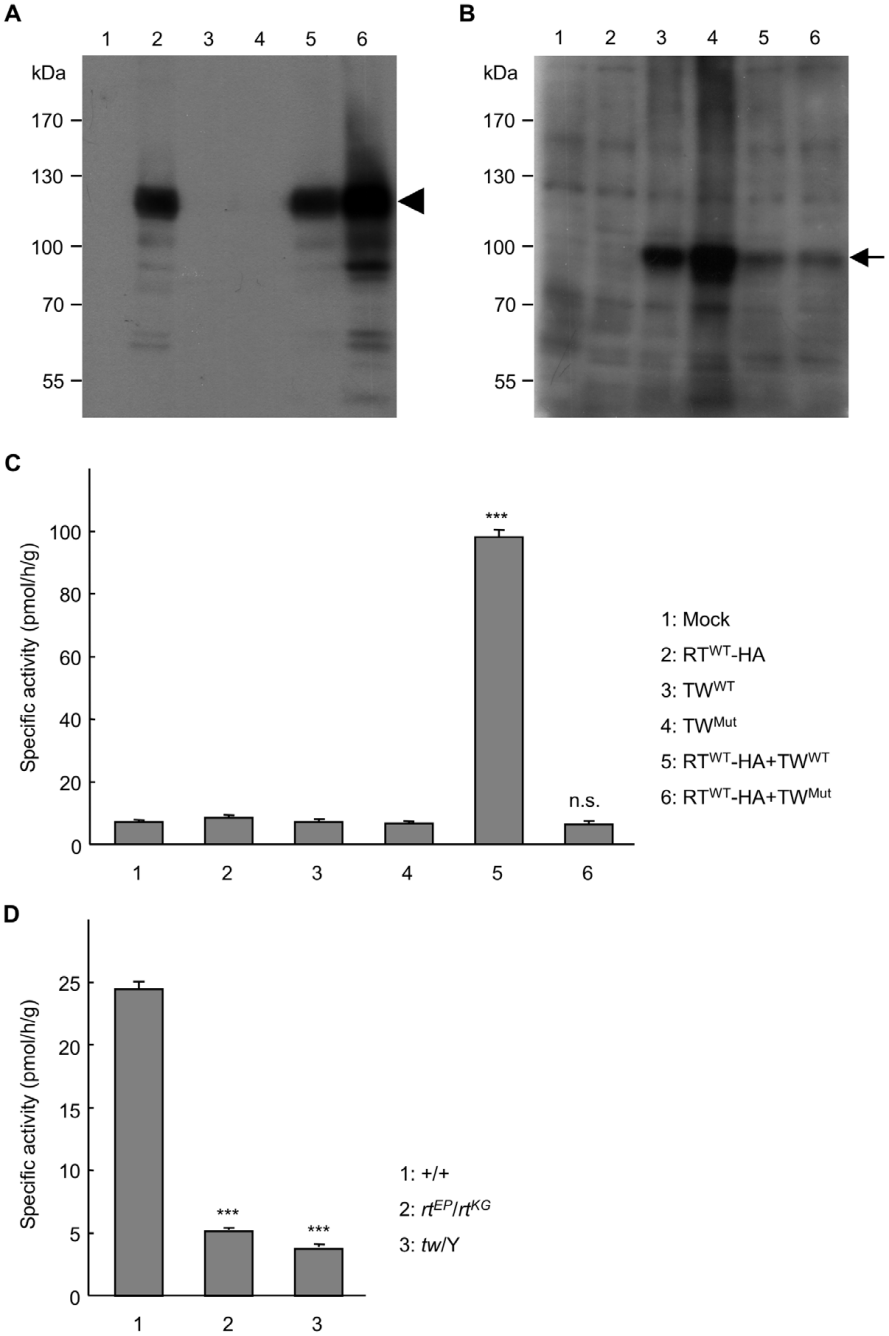
POMT activity of recombinant wild-type RT and wild-type or mutant TW flies. Western blot analysis of HA-tagged wild-type RT using anti-HA monoclonal antibody (A) and of TW using anti-TW antibody (B). The prepared microsomal membrane fractions of infected cells were applied to 7% SDS-PAGE at 20 µg for western blot analysis with anti-HA monoclonal antibody and 5 µg for that with anti-TW antibody. The arrowhead and arrow indicate HA-tagged RT and TW, respectively. (C) POMT activity for GST-α-DG of recombinant mutant TW (TW^Mut^). POMT-specific activity was detected when RT^WT^-HA and TW^WT^ were co-expressed as we reported. However, POMT-specific activity was not detected when RT^WT^-HA and TW^Mut^ were co-expressed. Each bar represents the mean of 8 replicates. Error bars indicate standard error. ***p<0.001 by the Tukey test. n.s, not significant. (D) POMT activity for GST-α-DG of larval extract of *rt* and *tw* mutant. POMT-specific activities of *rt* and *tw* mutant were significantly reduced. Each bar represents the mean of 6 replicates. Error bars indicate standard error. ***p<0.001 by *t* test.

Since there is no evidence that *POMT2* mutation in WWS patients influences POMT activity, we examined the POMT activity in the *Drosophila POMT2* mutant. We prepared recombinant wild-type RT (RT^WT^), wild-type TW (TW^WT^), and mutant TW (TW^Mut^) in order to examine POMT activity in the *tw* mutant. After pVL1393-*rt^WT^*-*HA* and pVL1393-*tw^Mut^* or pVL1393-*tw^WT^* was co-transfected into Sf21 insect cells, microsomal membrane fractions were collected from each infected cell. The specific expression of recombinant proteins was confirmed by western blot analysis using anti-HA monoclonal antibody and anti-dPOMT2 antibody (Figs. 12A and B). POMT activity toward GST-α-DG was measured using each microsomal membrane fraction as described under “Materials and Methods.” We could not detect enzymatic activity when only RT^WT^-HA, TW^WT^, or TW^Mut^ was expressed. POMT-specific activity was detected when RT^WT^-HA and TW^WT^ were co-expressed (*p*<0.001, Tukey test). However, no POMT-specific activity was detected when RT^WT^-HA and TW^Mut^ were co-expressed (n.s., Tukey test) (Fig. 12C). These data clearly demonstrated that the mutation involved in the *tw* mutant was a null mutation for POMT activity. Moreover, we examined the POMT activity of larval extracts of *rt* and *tw* mutants. POMT-specific activities of the *rt* and *tw* mutants were significantly reduced compared to those of wild-type flies (Fig. 12D). Taken together, the *tw* mutant was a null mutant and the *rt* mutant we used was an almost null or a strong hypomorphic mutant for POMT activity.

### Genetic interaction between *rt* or *tw* and *Dg* in the wing

**Figure 13:**
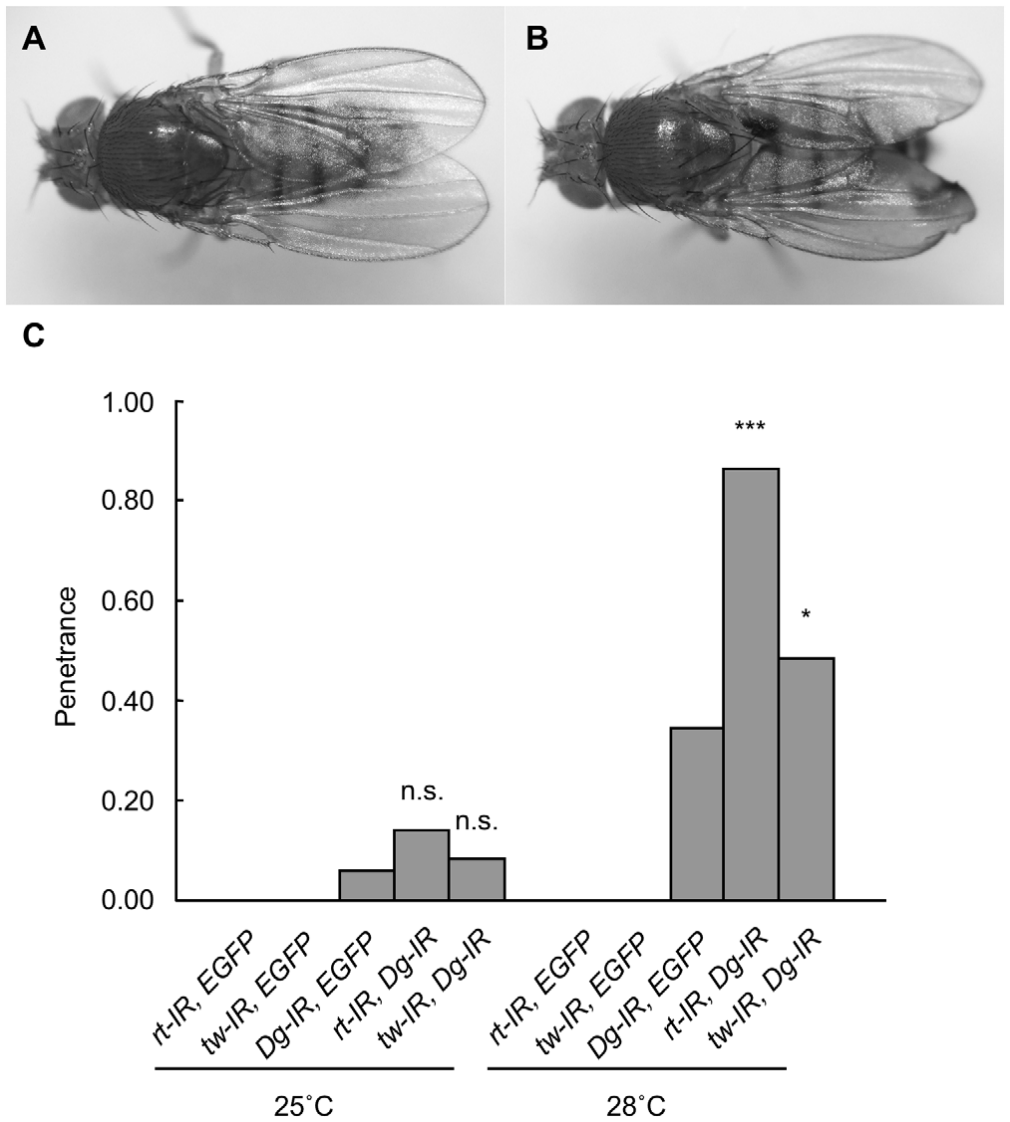
Genetic interaction between *rt* or *tw* and *Dg* in the wing. (A) Normal wing shape in wild-type flies. (B) Blistered phenotype in the wings of knockdown flies. (C) Penetrances of the blistered phenotype in knockdown flies. At least 30 individuals were observed in each knockdown group. At 28°C, the penetrances of the blistered phenotype with the double knockdowns *rt*-*Dg* and *tw*-*Dg* were significantly higher than those with single knockdown. *p<0.05; ***p<0.001 by Fisher's exact test. n.s., not significant.

Dg is one of the putative core proteins that are *O*-mannosylated by RT and TW. Wild-type flies had normal-shaped wings (Fig. 13A); however, knockdown of *Dg* in the posterior region of the wing disc resulted in a blistered phenotype (Fig. 13B). The blistered phenotype results from cell adhesion failure in the 2 cell layers during wing development. Actually, Dg expression was dramatically decreased in the posterior region (Fig. S5), indicating that Dg contributed the attachment of the 2 cell layers during wing development. We examined the genetic interaction between *rt* or *tw* and *Dg* by using this phenotype. At 25°C, the penetrances of the blistered phenotype in wings of single knockdown flies of *rt*, *tw*, and *Dg* were 0, 0, and 0.06, respectively. The penetrances of the phenotype in double knockdown flies of *rt-Dg* and *tw-Dg* were 0.14 and 0.08, respectively, but they were not significantly higher than those of the single knockdown flies (Fisher's exact test). As mentioned in the above section, “Lethality in *tw* mutants and flies expressing RNAi for the *rt* gene,” knockdown at 28°C is more efficient than that at 25°C. Thus, we performed the knockdown at 28°C. At 28°C, the penetrances of the blistered phenotype in wings of single knockdown flies of *rt*, *tw*, and *Dg* were 0, 0, and 0.34, respectively. The penetrances of double knockdown flies of *rt-Dg* and *tw*-*Dg* were 0.86 and 0.48, and they were significantly higher than those of single knockdown flies (*p*<0.001 and *p*<0.05, Fisher's exact test) (Fig. 13C). These data showed that *rt* or *tw* genetically interact with *Dg* to contribute to cell adhesion in the wings. Together with the high density of myoblasts observed in the *tw* mutant (Fig. 7F), these results suggest that both epidermal cells and muscle progenitor cells of *Drosophila POMT* mutants give rise to cell adhesion derangement.

## Discussion

In this study, we first presented not only the behavioral abnormalities but also the shortened lifespan and ultrastructural defects of muscles in flies with mutations in *rt* and/or *tw*, the *Drosophila* orthologs of human *POMT1* and *POMT2*, respectively. Our data strongly indicate that these mutants are a *Drosophila* model for WWS. We then discovered that apoptosis is enhanced in muscle progenitor cells of these mutants and provided new insight into the mechanism of WWS development, namely increased numbers of apoptotic myoblasts causing muscle disorganization.

### Behavioral and developmental similarities between the phenotypes of *rt* and *tw* mutant flies and the symptoms of WWS patients

The climbing abilities of *rt* and *tw* mutant flies were reduced compared to those of flies that were heterozygous for *rt* and *tw*, respectively (Figs. 1A and B, Tables 1 and 2). Reduced climbing ability was also observed in flies with ubiquitous expression of RNAi for the *rt* gene driven by *Act5C-Gal4* (Fig. 2, Table 3). In addition, *Dg* and *Dys* (*Dystrophin*) mutant flies lack climbing ability [24]. These data indicate that the *O*-mannosyl glycan on Dg contributes to motor functions such as climbing. The mutation of *rt* or *tw* in *Drosophila* causes the behavioral defect like WWS, since the defect of *O*-mannosyl glycan on Dg leads to WWS [14], [22]. Furthermore, the climbing abilities of mutants rapidly decreased with age (Figs. 1A and B). These changes are similar to the behaviors seen in patients with WWS, such as difficulty in walking with age.

Both our study (Fig. 3) and other studies [25], [31] have revealed structural defects in the larval body wall muscles in *Dg*, *rt*, and/or *tw* mutants. Moreover, we observed various kinds of defective ultrastructural phenotypes in the thoracic and leg muscles of adult *tw* mutant flies (Figs. 4 and 5, and Table 4) that have been reported in muscle biopsies of WWS patients [36]. A recent study revealed that expression of GluRIIB, a subunit of the postsynaptic glutamate receptor, and the efficacy of synaptic transmission decreases at the neuromuscular junctions of larval *Dg* and *rt* mutants [26]. Muscle contraction and membrane resistance in larval body wall muscles changed in flies expressing RNAi for the *Dg* gene [25]. These changes might cause decreased motor function in adult *rt* and *tw* mutants. On the other hand, we observed no difference in locomotive activity between mutant and control flies (Figs. 1C and D). The mutant flies showed abnormalities in heavy exercises, such as climbing or flying, but not in light movements, such as locomotion, probably because some muscles and/or neuromuscular junctions had normal functioning while other muscles had defective functioning.

The growing of flies with ubiquitous expression RNAi for the *rt* gene at a higher temperature resulted in higher lethality rates, and knockdown at 28°C was almost entirely lethal (Table 8). Some *rt* alleles that were hemizygous for the deficiency and entirely lacked the genomic region of *rt* showed partial lethality [31]. Thus, a null mutation in *rt* appears to be lethal. On the other hand, the *tw^1^* allele is semi-lethal (Table. 6). Davis [37] also reported that flies carrying a *tw* mutation showed reduced viability. Furthermore, we rescued the lethality of *tw* mutant flies by ubiquitous expression of the *tw* gene (Table 7). The apparent incidence rate of WWS is very low [10] because of its high lethality rate in the embryonic stage. Indeed, defective development in the early embryonic stages causes embryonic lethality in *Pomt1* mutant mice [23]. However, the mechanism underlying this lethality is unknown. Thus, the mechanism of the high lethality rate in the embryonic stage in mammals may be understood by elucidating the mechanisms of the high lethality rates in *rt* and *tw* mutant flies. Lethality in *rt* mutant flies is not associated with any particular developmental stage [31]. Several studies have revealed that *Dg* is involved in epithelial and oocyte polarity determination [38]–[40], so the *O*-mannosyl glycans on Dg at least contribute to the viability through fly oocyte formation and motor function.

Even though WWS patients may survive to birth, their lifespan is generally short and they typically die before reaching adulthood. Flies with ubiquitous expression of RNAi for the *rt* gene have shortened lifespans as well, but those with neuron- or glial cell-specific expression of RNAi do not have shortened lifespans (Fig. 11, Tables 9 and 10). These results suggest that expression of *POMT1* and *POMT2* in the tissues other than neurons and glial cells plays a crucial role in longevity in humans.

### Anatomical similarities between the muscles of *tw* mutant flies and WWS patients

In the present study, we showed that the larval body wall muscles (Fig. 3A, muscle 5) were thin or missing in flies expressing RNAi for the *rt* and *tw* genes as in flies carrying other mutant alleles (Figs. 3B–D). Furthermore, we observed living larval body wall muscles in *tw* mutant flies by using the *MHC*-*tauGFP* reporter, a muscle marker (Figs. 3F–H). Studies have shown that larval body wall muscles of *rt* and *tw* mutant flies are sometimes thin and missing [25], [31]. In our analysis, the frequency of abnormal patterning in the body wall muscles is approximately 50% in female and 40% in male *tw* mutant flies, both of which are higher than that reported in one study (10%) [25]. The discrepancies between the data seem to result from differences in the methods used: our methods did not involve dissection of larvae but, rather, observation of all muscles in living larvae using *MHC*-*tauGFP* reporter for muscle visualization; on the other hand, methods used to obtain the previous data included dissection, fixation, FITC-phalloidin staining for muscle visualization, and observation of particular muscles.

The ultrastructure of muscles in adult mutant flies has not yet been reported, although age-dependent muscle degeneration and large sarcomeres in the larval body wall muscles of the flies in mutants have been reported based on light microscopic observations [24], [25]. Here, we observed ultrastructural characteristics of *tw* mutant flies and discovered various phenotypes: sarcomeric disarray, irregular Z-lines, filament disorganization, swollen SR, accumulation of glycogen granules, enlargement of mitochondria, and duplication of basement membranes (Figs. 4 and 5). The ultrastructural defects observed in the thorax and leg muscles of *tw* mutant flies are consistent with muscle characteristics of patients with Duchenne muscular dystrophy, including WWS. Myofibril shearing, myofilament loss, wavy or disrupted Z-lines, changes in the size of SR and mitochondria, and clumped glycogen particles were observed in the muscles of patients in whom Duchenne muscular dystrophy was diagnosed clinically, histologically, and by serum creatine kinase assay [36]. In addition to the similarities in ultrastructural muscle defects seen between humans and flies, we demonstrated that the muscle phenotype increases in severity with age in flies (Fig. 6 and Table 5). These facts suggest that both the muscle ultrastructure and the function of *rt* and *tw* in maintaining the muscle structure are conserved in flies and humans.

### Similarities between POMT activities of WWS patients and *tw* mutant flies

POMT activity was clearly detected in Sf21 cells that co-expressed RT^WT^ and TW^WT^ but could not be detected in cells that expressed only RT^WT^ or TW^WT^ (Fig. 12). We obtained similar results previously [29]. In humans, coexistence of POMT1 and *POMT*2 is required for POMT activity [18], just as both RT and TW are required in flies. Mutations in the *POMT1* gene considered to cause WWS lead to reduced POMT activity and a defect in protein *O*-mannosylation [22].

However, POMT activity in WWS patients carrying a *POMT2* mutation has never been reported. We report here for the first time that a mutation in the *tw* gene, the *Drosophila* ortholog of *POMT2*, causes a reduction in *O*-mannosyltransferase activity (Fig. 12) and thus results in a defect in protein *O*-mannosylation. TW^WT^ protein has 7 transmembrane helices, and the TW^Mut^ protein (T59GS) contains a change in the first transmembrane region as predicted by SOSUI (http://bp.nuap.nagoya-u.ac.jp/sosui/), a secondary structure prediction program. These results indicate that the first transmembrane region of TW may play an important role in *O*-mannosyltransferase activity. Other regions in the *POMT2* gene important for POMT activity will be found through study of the mutations, enzymatic activity, and secondary structure of the protein in WWS patients, who have further *POMT2* gene mutations.

### New functional aspect of *POMTs* obtained from analyses of *rt* and *tw* mutant flies

CMDs, including WWS, cause progressive muscle degeneration and necrosis [41]. These muscle changes result from a defect in the DGC, including the *O*-mannosyl glycan, which is synthesized by POMTs [22]. DGC plays an important role in binding between the cytoskeleton and basal membrane [42], [43]. Because muscles are always subjected to severe physiological conditions, fragile binding between the cytoskeleton and basal membrane in patients with muscular dystrophies seems to result in weakness of the plasma membrane in muscles and degeneration of muscle fibers. Our results regarding the behavior and muscle structure of *rt* and *tw* mutant flies indicate that the *rt* and *tw* genes maintain muscle structure. Actually, the basal membrane was duplicated in muscles of the *tw* mutant (Figs. 4J and 5H). Further, *O*-mannosyl glycans on Dg contributed to attachment of the wing cells (Fig. 13). The above results suggest that cell adhesion in both muscle and epithelial cells, such as wing cells, which is involved in the *O*-mannosyl glycans on Dg, contributes to tissue or organ organization in *Drosophila POMT* mutants.

Interestingly, we showed that apoptosis was increased in myoblasts of wing imaginal discs in *tw* mutant larvae, cells that otherwise differentiate into indirect flight muscles (Fig. 8G). Myoblast density was observed to be high (Fig. 7F), although no mitotic abnormality was observed (Fig. S4). Moreover, expression of α-spectrin and βPS-integrin in the myoblasts was increased (Fig. 9). These results suggest that myoblasts of the *tw* mutant might avoid enhanced apoptosis or compensate for a DCG defect by overexpressing cytoskeletal or cell adhesion molecules such as α-spectrin and βPS-integrin. Mutations in the *tw* gene will lead to a high density of myoblasts, cause disruption of myoblast intercellular interactions, and result in enhanced myoblast apoptosis. Thus, the *tw* gene also controls myoblast density or intercellular interaction.

Here, we propose a novel mechanism for the development of WWS. At first, *POMT* mutation causes cell adhesion abnormalities, myoblast position derangement, and a high density of myoblasts because *O*-mannosyl glycans do not form on core proteins, such as Dg. Since high densities of myoblasts do not develop into normal muscles, some of them are excluded by apoptosis. As a result, muscles in the *POMT* mutant have duplicated basal membranes and decreased motor function compared to those of wild-type flies. Apoptosis occurs not only in myogenesis but also in cell differentiation events such as neurogenesis. Thus, severe phenotypes will appear in several organs of patients with WWS.

### Genetic interaction between *Dg* and *rt* and/or *tw* in the wing

Several studies have revealed the genetic interaction between *Dg* and *rt* and/or *tw*. One study demonstrated that *Dg* and *rt* contribute to the promotion of synaptic vesicle release and regulate glutamate receptor subunit composition at the neuromuscular junction [26]; moreover, it showed that muscle attachment formation and sarcomere size determination in third instar larvae require functional *Dg* and *rt* or *tw* [26].

In the present study, we showed that basement membranes are duplicated and multilayered in *tw* mutant muscles (Figs. 4J, 5H). This change in the basement membrane is due to the muscle attachment failure that results from mediation of Dg and laminin by *O*-mannosyl glycan [43]. In addition, knockdown of *Dg* in the wing resulted in the blistered phenotype (Fig. 13B), which was caused by cell adhesion failure of the 2 cell layers during wing development. Moreover, double knockdown of *Dg* and *rt* or *tw* enhanced this phenotype (Fig. 13C). Consequently, we demonstrated that the effect of *O*-mannosyl glycan on Dg contributes to epidermal cell attachment in the wing. Incidentally, a mutant of *wb* (*wing blister*), a *Drosophila* laminin, also showed blistered wings [44]. Thus, it is suggested that *O*-mannosyl glycan mediates the binding of Dg with molecules such as laminin in the extracellular matrix, and that failure of this binding leads to the blistered wing phenotype observed in mutants. Laminin and adhesion molecules play important roles in muscle attachment [45]–[47]. Thus, the binding of Dg to laminin mediated by *O*-mannosyl glycan might contribute to adhesion between the 2 epithelial cell layers of the wing as well as muscle attachment.

### Conclusion

Analyses of *rt* and *tw* mutants, the *Drosophila* models for WWS, help in understanding not only the symptoms of this human disease but also the mechanisms of muscular dystrophy. We proposed a new mechanism for the development of muscular dystrophy involving increased apoptosis in developing muscles that causes muscle disorganization. Further studies with these mutants will provide additional insight into the mechanisms of muscular dystrophies and will help in the development of useful drugs for WWS patients.

## Materials and Methods

### Fly stocks

All stocks were raised at room temperature (23–25°C) using a cornmeal-yeast-glucose medium. All stocks except those for *rt^EP571^*, *MHC*-*tauGFP*, *1151*-*Gal4*, and Canton-S were obtained from Bloomington Stock Center (http://flystocks.bio.indiana.edu/). We refer to the *rt^KG04772^* line (stock number: 14434) as *rt^KG^*. *rt^EP571^* was obtained from Szeged Stock Center. In this paper, we refer to this line as *rt^EP^*. *Df(1)Exel6223*, which disrupts the *tw* gene, is referred to as *Df(1)*. The *tw* stock was backcrossed onto the Canton-S background for 20 generations. *MHC*-*tauGFP* [48], a muscle marker, and *1151*-*Gal4* [49], a driver for marking the myoblasts in wing imaginal discs, were supplied by Dr. T. Maqbool. Canton-S was a gift from Dr. D. Yamamoto. *UAS*-*tw*-*IR* and *UAS*-*rt*-*IR* have been referred to by Ichimiya et al. [29] as *UAS*-*dPOMT2*-*IR* and *UAS*-*dPOMT1*-*IR*, respectively. *UAS*-*Dg*-*IR* and *UAS*-*tw* were generated by the following methods.

### *UAS*-*Dg*-*IR* flies

The *UAS-IR* fly line was obtained as described in previous reports [29], [32]. The cDNA fragment of *Dg* (nucleotide positions 327–826 of the coding sequence of *Dg*-*RA*) was amplified by PCR using a cDNA library derived from *Drosophila melanogaster*.

The amplified fragment was inserted as an inverted repeat (IR) sequence into the pSC1 vector. The IR-containing fragment was then subcloned into the transformation vector pUAST, and this vector was introduced into *Drosophila* embryos of the *w* ^1118^ mutant stock, which was used as the host to construct the *UAS*-*IR* fly line according to the procedure reported by Spradling and Rubin [50].

### *UAS*-*tw* flies

The DNA fragment containing the *tw* gene was amplified by two-step PCR. For the first PCR, we used the plasmid DNA from an EST clone (LP01681) as a template and the following primer set for the amplification of the *tw* coding region: forward primer 5′-AAAAAGCAGGCTTGGCAGCAAGTGTTGTTA-3′; and reverse primer 5′-AGAAAGCTGGGTCTAGAACTCCCAGGTAGAAAG-3′. For the second PCR, we used the first PCR product as a template; a forward primer that included the attB1 site, 5′-GGGGACAAGTTTGTACAAAAAAGCAGGCT-3′; and a reverse primer that included the attB2 site, 5′-GGGGACCACTTTGTACAAGAAAGCTGGGT-3′. The amplified fragments were subcloned into the pDONR201™ vector (Invitrogen, http://www.invitrogen.com). The inserted fragment was then recombined between the attR1 and attR2 sites in the multi-cloning site of the modified pUAST vector to yield pUAST-*tw*. The transgenic fly was generated by the method described in the “*UAS*-*Dg*-*IR* flies” section.

### Climbing assay

We used a modified version of a previously described assay to assess climbing ability [51]. Twenty individual flies were gently introduced into a glass vial height 240 mm tall and 25 mm in diameter. After a 5-minute rest, the bottom of the vial was gently tapped and the maximum height reached in 10 seconds was recorded by a digital camera. Five trials were performed in each experiment. Climbing ability was then calculated from the 5 trials.

### Locomotion assay

We used the procedure described in Kaneuchi et al. to assess locomotive ability [52]. One day after eclosion, flies were individually introduced into a glass tube 65 mm long and 3 mm in diameter with a medium containing 60% swelling SP-Sephadex C-50 (weight/volume), 10% glucose, 0.6% propionic acid, 2% yeast extract, and 1.2% agar. After the tubes were placed in the Drosophila Activity Monitor (TriKinetics Inc., http://www.trikinetics.com/), the medium was changed every week until the fly died. The number of times a fly crossed the center of the glass tube was automatically recorded every 30 minutes. At least 16 individuals were tested in each group.

### Flight assay

We used a modified version of the method by Stockinger et al. to assess flying ability [53]. Twenty individual flies (30–35 days of age) were dumped through a plastic funnel into a glass cylinder 450 mm in height and 80 mm in diameter whose inside surface was coated with mineral oil. Numbers on the y-axis represented the height marks on the glass cylinder: 1, 0–200 ml; 2, 200–400 ml; 3, 400–600 ml…10, 1800–2000 ml. Scores were individually recorded, and the score average was calculated from 5 independent experiments.

### Longevity assay

About 30 test flies were maintained in the standard medium after eclosion for assessment of longevity. Every 2 or 3 days, flies were transferred to a fresh food vial and the number of dead flies was determined until all flies died. More than 4 replicate vials were tested in each group and data were pooled for statistical analysis.

### Staining of muscles and examination of the frequencies of abnormal muscle patterning

Third instar larvae were dissected along the dorsal midline in ice-cold phosphate-buffered saline (PBS: 130 mM NaCl, 7 mM Na_2_HPO_4_, and 3 mM NaH_2_PO_4_). After removal of the digestive organs, fat bodies, and main trachea, the preparations were fixed with 4% formaldehyde in PBS for 30 minutes at room temperature. The tissue was then washed in PBS with 0.1% Triton X-100 (PBT) and stained with phalloidin conjugated with FITC. Stained samples were mounted in 90% glycerol in PBS and observed under a Zeiss LSM5 Pascal confocal microscope. We counted the number of individuals that had more than 1 abnormal muscle and calculated the frequencies of abnormal muscle patterning.

### Immunohistochemistry

Third instar larvae were dissected in ice-cold PBS. Wing imaginal discs were fixed with 4% paraformaldehyde (pH 7.0) in PBS for 15 minutes and washed 3 times with PBT. After being blocked with 10% goat serum in PBT, the samples were stained with primary antibodies. The primary antibodies were used in the following dilutions: anti-cleaved caspase-3 (Asp175) rabbit polyclonal antibody, 1∶300 (Cell Signaling, http://www.cellsignal.com); anti-phospho-histone H3 (Ser10) rabbit polyclonal antibody, 1∶100 (Millipore, http://www.millipore.com); anti-α-spectrin mouse monoclonal antibody (3A9), 1∶25 (Developmental Studies Hybridoma Bank, http://dshb.biology.uiowa.edu/); anti-βPS-integrin mouse monoclonal antibody (CF.6G11), 1∶200 (Developmental Studies Hybridoma Bank); anti-DE-cadherin rat monoclonal antibody (DCAD2), 1∶25 (Developmental Studies Hybridoma Bank); and anti-Dg rabbit polyclonal antibody, 1∶100 [54]. The secondary antibodies used were anti-rabbit Alexa 594, anti-rabbit Cy5, anti-mouse Cy3, anti-mouse Cy5, or anti-rat Alexa 647 (Invitrogen). Stained samples were mounted in FluoroGuard™ Antifade Reagent (BIO-RAD, http://www.bio-rad.com) and observed under a Zeiss LSM5 Pascal confocal microscope.

### Electron microscopy and analysis of ultrastructural phenotypes

Legs and thoraces were isolated from adult flies (15 and 35 days old, males and females, n = 6) and fixed with 2.5% glutaraldehyde in PBS overnight at room temperature. They were then postfixed with 1% OsO_4_ in 100 mM phosphate buffer (pH 7.3) for 1 hr at 4°C and dehydrated in a graded series of alcohol. After passage through propylene oxide, the specimens were embedded in Epon 812. Ultrathin sections were cut, stained with uranyl acetate and lead citrate, and observed with a JEM-1010C transmission electron microscope (JEOL, http://www.jeol.co.jp). We counted the numbers of sarcomeric disarrays, irregular Z-lines, and filament disorganizations in a 590 µm^2^ area of muscle per individual and calculated the percentage of abnormal structures in the muscle.

### Quantitative analysis of the *rt* and *tw* transcripts in flies by real-time PCR

Total RNA was extracted from third instar larvae of each fly by using TRIzol® Reagent (Invitrogen). First-strand cDNA was synthesized using a SuperScript® II First-Strand Synthesis Kit (Invitrogen). Real-time PCR was performed using QuickGoldStar qPCR MasterMix (Eurogentec, http://www.eurogentec.com/) and the ABI PRISM® 7700 Sequence Detection System (Applied Biosystems, http://www3.appliedbiosystems.com/). The gene-specific primer pairs and TaqMan probes were used for each gene are as follows. For *rt* quantification, the forward primer 5′-ACACCTGTGGCAACTGCTCTAC-3′, reverse primer 5′-ACTTATGGCATGCATCCATAGCT-3′, and probe 5′-ACGCCGGTCTCACCGATCGC-3′ were used. For *tw* quantification, the forward primer 5′-TTTCCGGCCTTGATCTTCAA-3′, reverse primer 5′-TGGGCAGAACCCTCAAAATG-3′, and probe 5′-TCCTTGCTGACGGGCGTTATGTACAACT-3′ were used. For *Ribosomal protein L32 (RpL32)* quantification, the forward primer 5′-GCAAGCCCAAGGGTATCGA-3′, reverse primer 5′-CGATGTTGGGCATCAGATACTG-3′, and probe 5′-AACAGAGTGCGTCGCCGCTTCA-3′ were used. The probes were labeled with reporter dye FAM and quencher dye TAMRA at the 5′- and -3′ ends, respectively. The relative amounts of the *rt* and *tw* transcripts were normalized to those of the *RpL32* transcripts in the same cDNA.

### Vector construction and expression of recombinant h-Dg proteins for the assay of POMT activity

Human α-dystroglycan (α-hDG), which was used as the substrate for the *O*-mannosylation reaction, was amplified using the forward primer that included the *Eco*RI site, 5′-GAATTCCCATCCAGGATCGTGCCA-3′, and the reverse primer that included the *Not*I site, 5′-GCGGCCGCTTAGGTAGCAACTGCAGTAGGC-3′. The amplified fragment up to the region of amino acids 335–421 was subcloned into pGEX-6P-1 (GE Healthcare, http://www.gehealthcare.com/), the *N*-terminal glutathione-*S*-transferase (GST) fusion vector. The pGEX-6P-1-*α-hDG* transformant of *Escherichia coli* BL21 (DE3) was cultured until the OD_600_ reached 1.2 at 20°C and then incubated with 0.1 mM IPTG at 20°C for 6 hr. The cells were sonicated with a PBS solution containing 0.5% *n*-octyl-β-d-thioglucoside (DOJINDO LABORATORIES, http://www.dojindo.com/), 1 mM dithiothreitol (DTT), and protease inhibitors (5 µg/ml pepstatin A, 2 µg/ml leupeptin, 2 µg/ml aproptin, 1 mM benzamidine-HCl, and 1 mM phenylmethylsulfonyl fluoride [PMSF]) and centrifuged; the supernatant was then applied to Glutathione-Sepharose™ 4B beads (GE Healthcare). The recombinant GST-fused α-hDG protein was eluted with a solution containing 50 mM reduced glutathione, 20 mM Tris-HCl (pH 8.0), 10 mM ethylenediaminetetraacetic acid (EDTA), and 0.5% *n*-octyl-β-d-thioglucoside. To remove the glutathione, the eluate solution was changed to a solution containing 20 mM Tris-HCl (pH 8.0), 10 mM EDTA, 2 mM 2-mercaptoethanol, and 0.5% *n*-octyl-β-d-thioglucoside using a PD-10 column (GE Healthcare).

### Vector construction and expression of mutant form of TW

The full-length ORFs of the wild-type *rt* form, wild-type *tw* form, and mutant *tw* form were expressed in insect cells as described previously [29]. One mutant in *tw*, *tw^1^*, has been reported. Sequencing of the *tw* gene in *tw^1^* mutant flies revealed 3 alterations. Two of these alterations do not cause amino acid substitutions. The third alteration, a 2-base substitution and 3-base insertion, is predicted to affect the translated protein sequence: the 59th threonine residue from the initiating methionine changes to glycine and serine residues [30]. Hereafter, we represent the wild-type *rt* form, wild-type *tw* form, and mutant *tw^1^* form as *rt^WT^*, *tw^WT^*, and *tw^Mut^*, respectively. The coding region of *tw^Mut^* was amplified from the cDNA of *tw* mutant flies using the same primer sets as that for *tw^WT^* [29], and the amplified fragment was inserted into the vector pVL1393 g. pVL1393-*rt^WT^-HA*, pVL1393-*tw^WT^*, and pVL1393-*tw^Mut^* were co-transfected with BD BaculoGold Linearized Baculovirus DNA (BD Biosciences, http://www.bdbiosciences.com) into Sf21 insect cells, and the cells were incubated for 7 days at 25°C to produce recombinant viruses. Sf21 cells were infected with each recombinant virus and incubated to express RT^WT^-HA, TW^WT^, and TW^Mut^ proteins.

### Preparation of cellular microsomal membrane fraction and assay of POMT activity

The microsomal membrane fraction was prepared and the POMT activity was assayed as described previously [29], [55] with some modification. The infected cells were first collected and washed twice in PBS. They were then suspended in a solution containing 10 mM Tris-HCl (pH 7.4), 1 mM EDTA, 250 mM sucrose, 1 mM DTT, and protease inhibitors (5 µg/ml pepstatin A, 2 µg/ml leupeptin, 2 µg/ml aproptin, 1 mM benzamidine-HCl, and 1 mM PMSF) and homogenized using a 1-ml Dounce homogenizer. After centrifugation at 900×*g* for 10 minutes, the supernatant was subjected to ultracentrifugation at 100,000×*g* for 1 hr. The pellet was suspended in 20 mM Tris-HCl (pH 8.0), 10 mM EDTA, 2 mM 2-mercaptoethanol, and 0.5% *n*-octyl-β-d-thioglucoside, and this suspension was used as the microsomal membrane fraction. The reaction mixture contained 20 mM Tris-HCl (pH 9.0), 10 mM EDTA, 2 mM 2-mercaptoethanol, 0.5% *n*-octyl-β-d-thioglucoside, 100 nM Dol-P-[^3^H]Man (133,200 dpm/pmol) (American Radiolabeled Chemicals, http://www.arcincusa.com/), 2.5 µg GST-α-hDG, and 80 µg of microsomal membrane fraction as the enzyme source in a total volume of 20 µl. After 1-hr incubation at 18°C, the reaction was stopped by addition of 100 µl of PBS containing 1% Triton X-100, and the reaction mixture was centrifuged at 10,000×*g* for 5 minutes. The supernatant was transferred, mixed with 400 µl of PBS and 20 µl of Glutathione-Sepharose™ 4B beads, rotated for 1 hr, and washed 3 times with PBS containing 0.2% Triton X-100. The radioactivity of the beads was measured using a liquid scintillation counter.

### Western blot analysis

Each microsomal membrane fraction was subjected to 7% sodium dodecyl sulfate polyacrylamide gel electrophoresis (SDS-PAGE). The membrane to which the separated proteins were transferred was probed with anti-HA mouse monoclonal antibody (1∶2000 dilution) (Santa Cruz Biotechnology, Inc., http∶//www.scbt.com/) or anti-dPOMT2 rabbit polyclonal antibody (1∶400 dilution) [29]. Each membrane was then reacted with HRP-conjugated secondary antibody and stained with Amersham™ ECL™ Plus (GE Healthcare).

### Preparation of larval extract and assay of POMT activity

Fourty third instar larvae of *rt^EP^*/*rt^KG^*, *tw*/Y, and wild-type (Canton-S) flies were homogenized in 20 mM Tris-HCl (pH 8.0), 10 mM EDTA, 2 mM 2-mercaptoethanol, and 0.5% *n*-octyl-β-d-thioglucoside with protease inhibitors (5 µg/ml pepstatin A, 2 µg/ml leupeptin, 2 µg/ml aproptin, 1 mM benzamidine-HCl, and 1 mM PMSF) (300 µl for every 40 larvae). The supernatant was obtained by centrifugation at 9,000×*g* for 10 minutes and used as larval extract. The same reaction mixture and conditions described in the “Preparation of cellular microsomal membrane fraction and assay of POMT activity” section above were used except for the following 2 modifications: (1) the amount of GST-α-hDG in the reaction mixture was 10 µg; and (2) the incubation time was 2 hr.

### Statistical analyses

All statistical analyses were performed using the public domain R program (http://www.r-project.org/).

## Supporting Information

Figure S1 Quantitative analysis of *rt* and *tw* mRNAs in *rt* mutant fly. *rt* (A)and *tw* (B) transcript levels of *rt^EP^*/*rt^KG^* mutant flies of third instar larvae were determined by real-time PCR. Error bars indicate standard error. Lines above the bars show compared groups by one-way ANOVA. *p<0.05 by the one-way ANOVA. n.s., not significant. * above the bar of *rt^EP^*/*rt^KG^* means *p*<0.05 by Tukey test.
(0.15 MB TIF)

Figure S2 Quantitative analysis of *rt* and *tw* mRNAs in the flies with the expression of RNAi for *rt* gene. *rt* (A)and *tw* (B) transcript levels of the flies with the expression of RNAi for rt gene of third instar larvae were determined by real-time PCR. The expression level of *rt* in *Act5C*>*rt*-*IR* was significantly reduced, while that in other lines was not significantly reduced. The expression level of *tw* was no different among all lines. Error bars indicate standard error. Lines above the bars show compared groups by one-way ANOVA. ***p<0.001 by the one-way ANOVA. n.s., not significant. * above the bar of *Act5C*>*rt*-*IR* means *p*<0.05 by Tukey test.
(0.18 MB TIF)

Figure S3 Quantitative analysis of *rt* and *tw* mRNAs in *MHC*-*tauGFP* fly. *rt* (A)and *tw* (B) transcript levels of *MHC*-*tauGFP* flies of third instar larvae were determined by real-time PCR. The expression levels of *rt* and *tw* did not differ between in *MHC*-*tauGFP* and wild type. Error bars indicate standard error. n.s., not significant by one-way ANOVA.
(0.13 MB TIF)

Figure S4 Dividing myoblasts in the wing imaginal disc of *tw* mutant larva. The number of myoblasts positive for phospho-histone H3 in the wing imaginal discs of wild-type and *tw* mutant larvae. The phosho-histone H3 is marker of dividing cells. The number of dividing myoblasts did not differ between *tw* mutant and wild-type larva. n.s., not significant by *t* test.
(0.10 MB TIF)

Figure S5 Reduced expression of Dg in the posterior region of the wing. (A–D) Wing imaginal discs in the third instar larvae of *en*-*Gal4*>*UAS*-*EGFP*, *UAS*-*Dg*-*IR* flies. All discs are anterior left, dorsal up. (A) Differential interference contrast (DIC) image of the wing disc. (B) The knockdown region of *Dg*, which is visualized by EGFP (green). The expression of *en*-*Gal4* is the posterior region of the wing. (C) The expression of Dg (red) decreases in the posterior region of the wing. (D) Marged image of (B) and (C). EGFP and Dg do not co-localize in the wing. Dg dramatically decreases in the knockdown region of *Dg*.
(5.81 MB TIF)
