# Supplementary figures and images for: Increased Apoptosis of Myoblasts in Drosophila Model for the Walker-Warburg Syndrome
Source: PLoS One. 2010 Jul 13;5(7):e11557. doi: 10.1371/journal.pone.0011557 (PMC2903483; doi:10.1371/journal.pone.0011557)

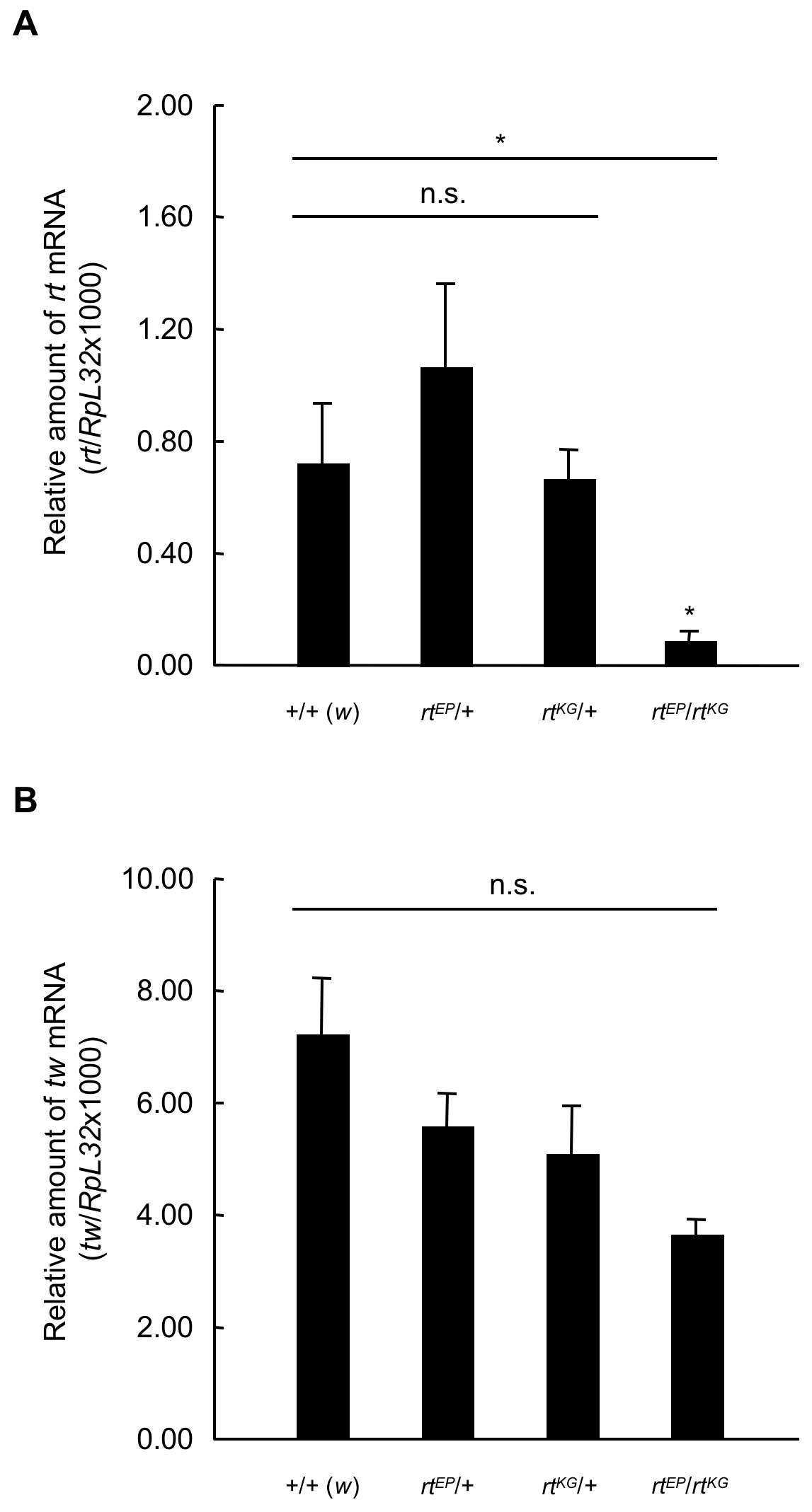

Supplement: Figure S1 — Quantitative analysis of rt and tw mRNAs in rt mutant fly. rt (A)and tw (B) transcript levels of rtEP/rtKG mutant flies of third instar larvae were determined by real-time PCR. Error bars indicate standard error. Lines above the bars show compared groups by one-way ANOVA. *p<0.05 by the one-way ANOVA. n.s., not significant. * above the bar of rtEP/rtKG means p<0.05 by Tukey test. (0.15 MB TIF) [file pone.0011557.s001.tif]

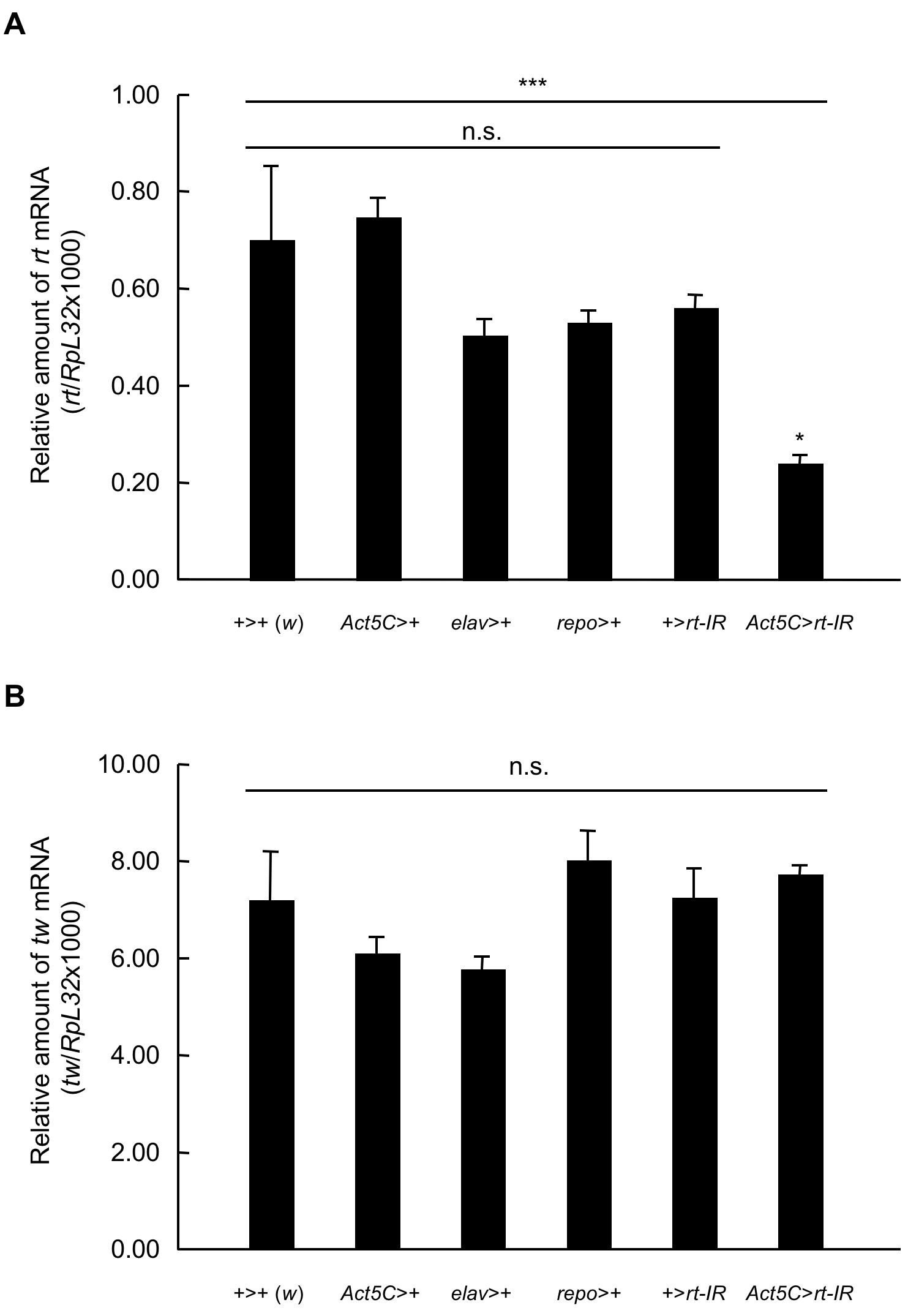

Supplement: Figure S2 — Quantitative analysis of rt and tw mRNAs in the flies with the expression of RNAi for rt gene. rt (A)and tw (B) transcript levels of the flies with the expression of RNAi for rt gene of third instar larvae were determined by real-time PCR. The expression level of rt in Act5C>rt-IR was significantly reduced, while that in other lines was not significantly reduced. The expression level of tw was no different among all lines. Error bars indicate standard error. Lines above the bars show compared groups by one-way ANOVA. ***p<0.001 by the one-way ANOVA. n.s., not significant. * above the bar of Act5C>rt-IR means p<0.05 by Tukey test. (0.18 MB TIF) [file pone.0011557.s002.tif]

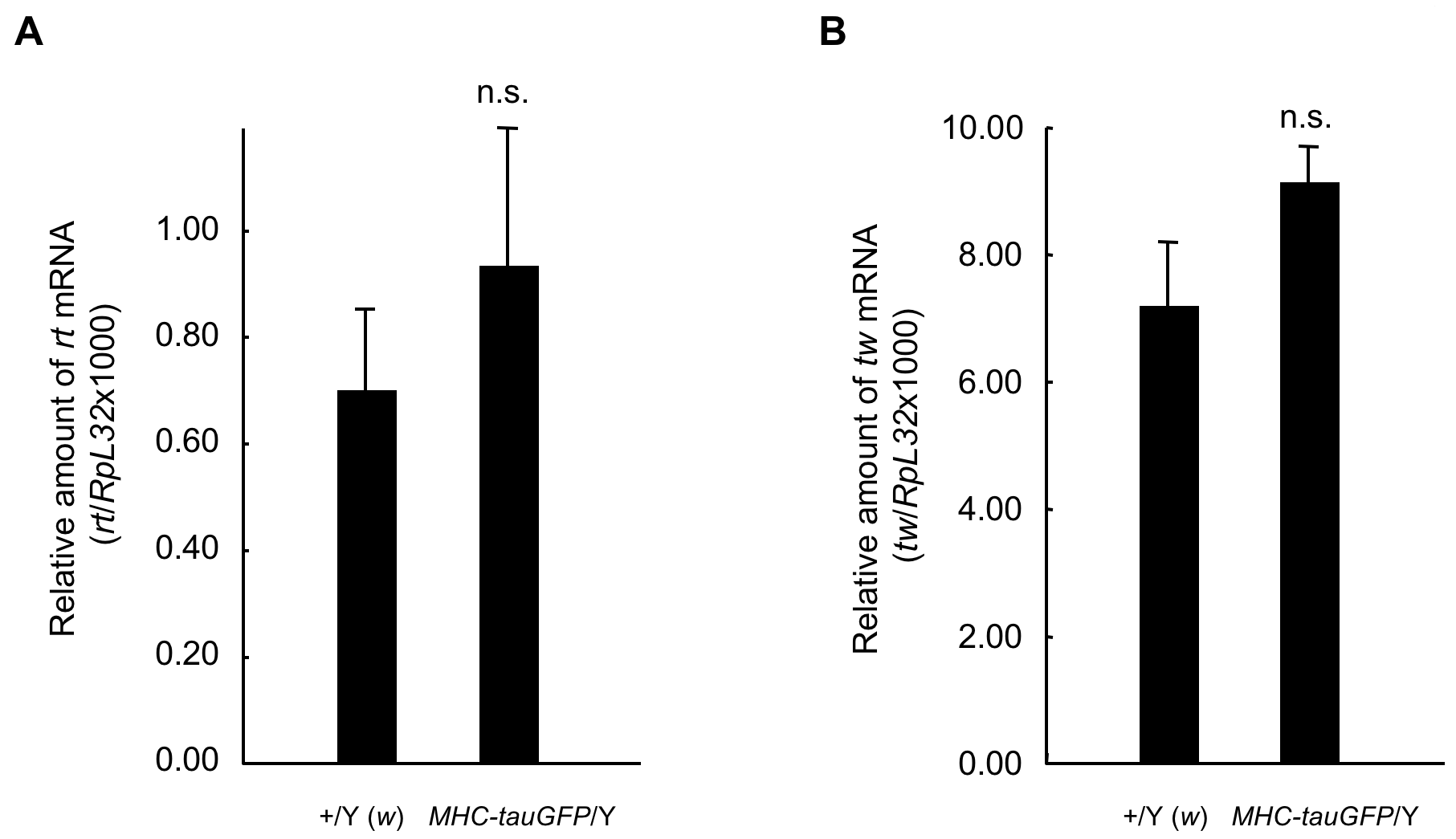

Supplement: Figure S3 — Quantitative analysis of rt and tw mRNAs in MHC-tauGFP fly. rt (A)and tw (B) transcript levels of MHC-tauGFP flies of third instar larvae were determined by real-time PCR. The expression levels of rt and tw did not differ between in MHC-tauGFP and wild type. Error bars indicate standard error. n.s., not significant by one-way ANOVA. (0.13 MB TIF) [file pone.0011557.s003.tif]

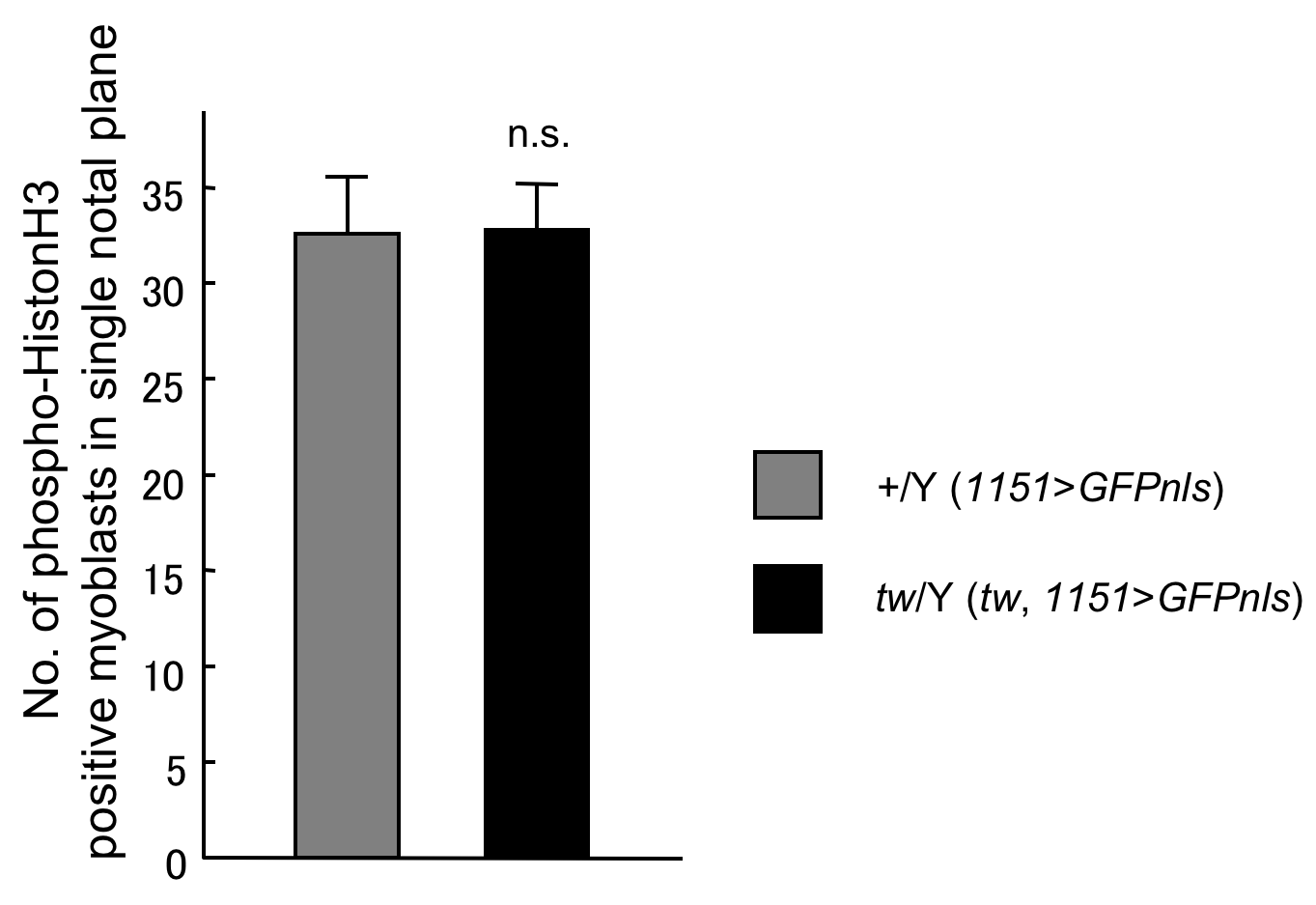

Supplement: Figure S4 — Dividing myoblasts in the wing imaginal disc of tw mutant larva. The number of myoblasts positive for phospho-histone H3 in the wing imaginal discs of wild-type and tw mutant larvae. The phosho-histone H3 is marker of dividing cells. The number of dividing myoblasts did not differ between tw mutant and wild-type larva. n.s., not significant by t test. (0.10 MB TIF) [file pone.0011557.s004.tif]

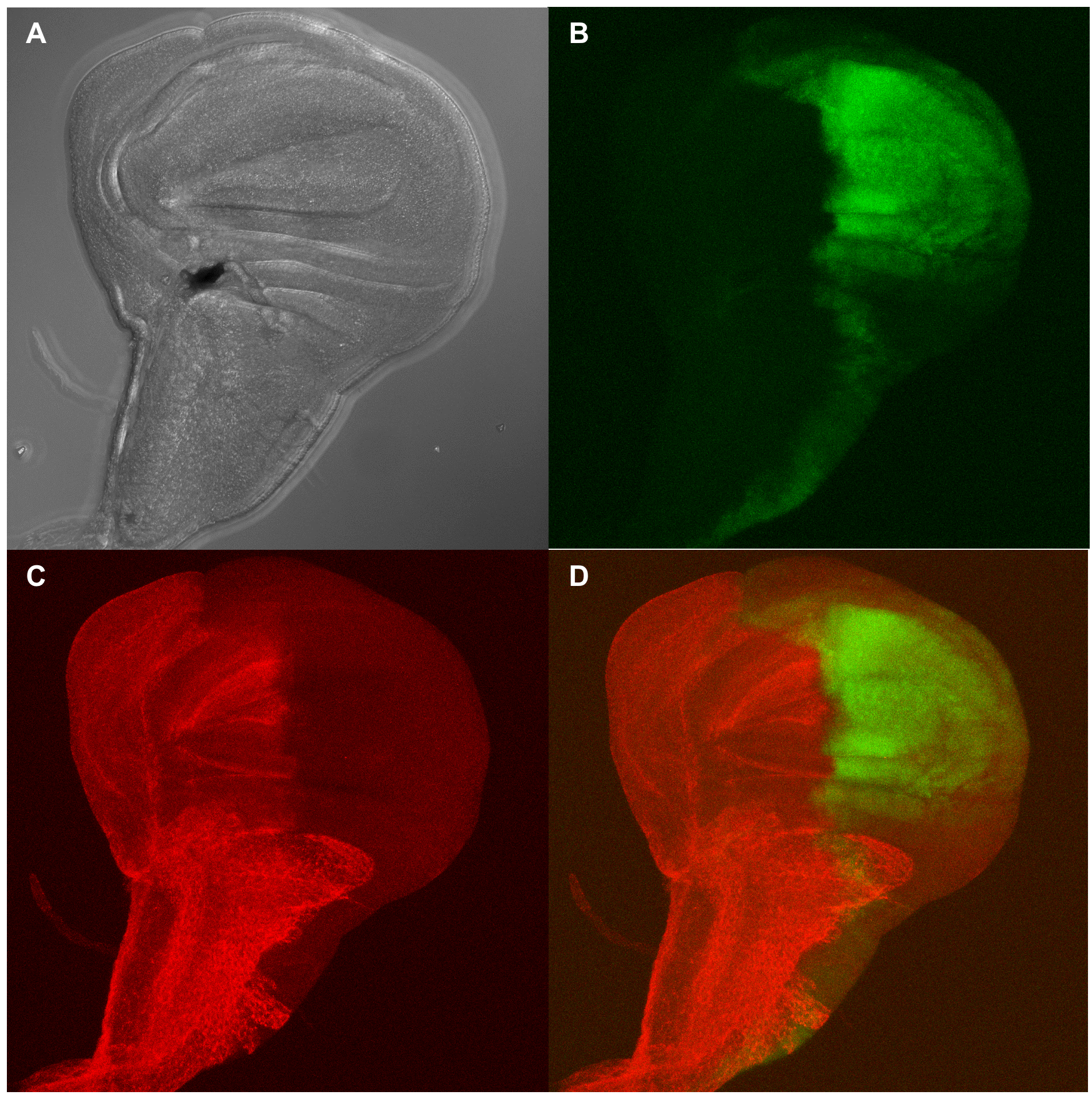

Supplement: Figure S5 — Reduced expression of Dg in the posterior region of the wing. (A–D) Wing imaginal discs in the third instar larvae of en-Gal4>UAS-EGFP, UAS-Dg-IR flies. All discs are anterior left, dorsal up. (A) Differential interference contrast (DIC) image of the wing disc. (B) The knockdown region of Dg, which is visualized by EGFP (green). The expression of en-Gal4 is the posterior region of the wing. (C) The expression of Dg (red) decreases in the posterior region of the wing. (D) Marged image of (B) and (C). EGFP and Dg do not co-localize in the wing. Dg dramatically decreases in the knockdown region of Dg. (5.81 MB TIF) [file pone.0011557.s005.tif]
